# Review of the Microstructural Impact on Creep Mechanisms and Performance for Laser Powder Bed Fusion Inconel 718

**DOI:** 10.3390/ma18020276

**Published:** 2025-01-09

**Authors:** Guillian Bryndza, Jérôme Tchoufang Tchuindjang, Fan Chen, Anne Marie Habraken, Héctor Sepúlveda, Víctor Tuninetti, Anne Mertens, Laurent Duchêne

**Affiliations:** 1 ArGEnCo Department, MSM Team, University of Liège, Quartier Polytech 1, Allée de la Découverte 9 (B52), 4000 Liège, Belgium; guillian.bryndza@uliege.be (G.B.); l.duchene@uliege.be (L.D.); 2 Department of Aerospace and Mechanical Engineering, MMS Team, University of Liège, Quartier Polytech 1, Allée de la Découverte 9 (B52), 4000 Liège, Belgium; j.tchuindjang@uliege.be (J.T.T.);; 3 Fonds de la Recherche Scientifique−F.R.S.−F.N.R.S. Belgium, 5 Rue d’Egmont, 1000 Brussels, Belgium; 4 Department of Mechanical Engineering, Universidad de La Frontera, Francisco Salazar 01145, Temuco 4780000, Chile

**Keywords:** additive manufacturing, LPBF, nickel-based superalloys, Inconel 718, creep, microstructure, heat treatment

## Abstract

Inconel 718 (IN718) is a polycrystalline nickel-based superalloy and one of the most widely used materials in the aerospace industry owing to its excellent mechanical performances at high temperatures, including creep resistance. Interest in additively manufactured components in aerospace is greatly increasing due to their ability to reduce material consumption, to manufacture complex parts, and to produce out-of-equilibrium microstructures, which can be beneficial for mechanical behavior. IN718’s properties are, however, very sensitive to microstructural features, which strongly depend on the manufacturing process and subsequent heat treatments. Additive manufacturing and, more specifically, Laser Powder Bed Fusion (LPBF) induces very high thermal gradients and anisotropic features due to its inherently directional nature, which largely defines the microstructure of the alloy. Hence, defining appropriate manufacturing parameters and heat treatments is critical to obtain appropriate mechanical behavior. This review aims to present the main microstructural features of IN718 produced by LPBF, the creep mechanisms taking place, the optimal microstructure for creep strength, and the most efficient heat treatments to yield such an optimized microstructure.

## 1. Introduction

**Figure 1:**
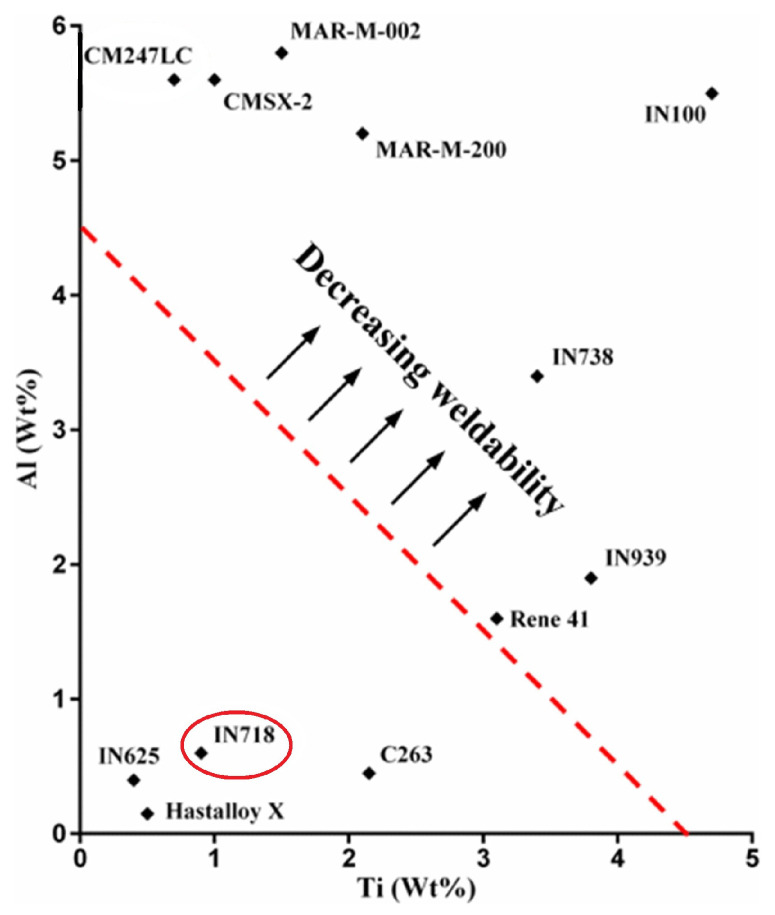
“Weldability” diagram for a range of Ni alloys as a function of their Ti and Al content. Weldability is considered poor above the dashed line and deteriorates with increasing Ti and Al content. Reproduced from Catchpole-Smith et al. [11].

Inconel 718, also referred to as IN718, Alloy 718, or GH4169, is a nickel-based superalloy extensively used in the aerospace, chemical, and power industries for its outstanding high-temperature mechanical properties. It exhibits excellent creep and fatigue performances at temperatures up to 650–700 °C [1,2,3,4] as well as superior corrosion and oxidation resistance [4,5]. As reported by Volpato et al. [6], it was the most widely used alloy in aerospace in 2022, both by weight fraction and market volume. It is also a prime candidate for Additive manufacturing (AM), such as Laser Powder Bed Fusion (LPBF, previously known as Selective Laser Melting or SLM), due to its good weldability [1,3,5,7]. This feature is attributed to its relatively slow kinetics of precipitation and good resistance to solid-state cracking [1,3,5], allegedly owing to its low content of Al and Ti [8,9,10,11], as shown in Figure 1. In addition, Xu et al. [12] highlighted that AM is particularly well-suited for aerospace applications, enabling the manufacturing of relatively small quantities of complex components. It also solves the issue of poor machinability inherent in high-strength alloys due to their low thermal conductivity, high hardness, and high shear strength [3,8]. Since most high-temperature components are designed to deform at a minimum creep rate under the identified service conditions [13,14], creep is a primary design criterion. In this respect, Ni-based superalloys are mostly used for their high-temperature strength, creep performance, and good temperature-dependent properties.

Despite the benefits of AM, our limited understanding of process-induced microstructural features and of the associated mechanical response of LPBF IN718 is prohibitive to its use for load-bearing parts, especially in the aerospace industry where standards are extremely high. Indeed, LBPF manufacturing is characterized by high thermal gradients, causing segregation of refractory elements such as Nb and Mo, promoting out-of-equilibrium detrimental phases and generating hierarchically inhomogeneous microstructures [15]. Although numerous articles are published every year regarding these features, no consensus has been reached on optimal AM processing parameters and subsequent heat treatments, unlike the established practices in conventional casting and forming methods. In addition, in their review paper dealing with real-time measurements on metal AM, Ioannidou et al. [16] described the existing instrumentation and the relevant synchrotron measurement capabilities. The phenomena that have been studied for IN718 based on such techniques correspond to Melt Pool (MP) and powder dynamics, phase transformation, stress, and temperature evolution. Nevertheless, only one study has been reported that deals with LPBF IN718; the other ones consider either Laser-Directed Energy Deposition (LDED) or Electron Beam Powder Bed Fusion (EBPBF) processes. Therefore, there is still work to be completed in the future on the real-time monitoring of phenomena occurring in LPBF IN718, which will help achieve a better understanding of the material behavior during both AM and subsequent post-treatments.

Moreover, despite their significance, the creep properties of Ni-based superalloys processed by LPBF are not studied enough, as depicted by Sanchez et al. [10]. They reported that only 7% of the 290 papers analyzed within their general review focused on creep. A similar trend was evidenced by Volpato et al. [6] in their review of the LPBF of Inconel superalloys.

**Figure 2:**
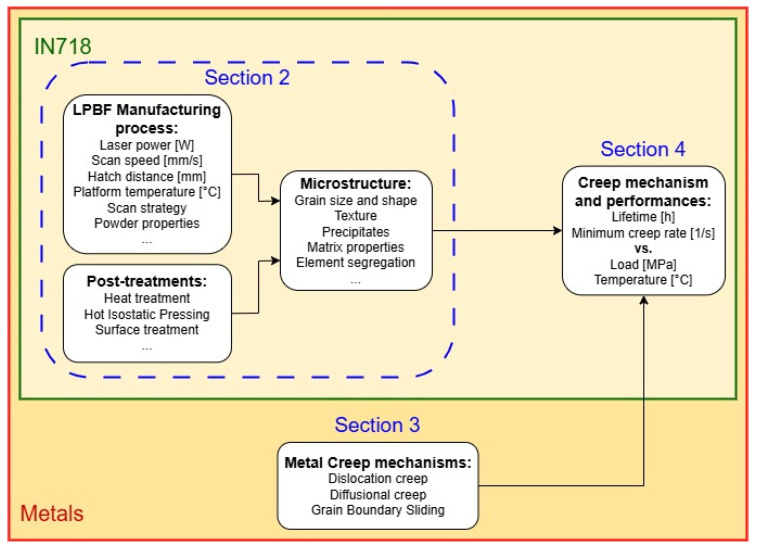
Flow chart describing the process–creep performance link.

Therefore, this review paper aims to understand and summarize the creep mechanisms of IN718 processed by LPBF to address the existing knowledge gaps on this topic. A secondary objective of this study is to correlate final properties to microstructure with the purpose of showing whether the mechanical performances of IN718 components at high temperatures can be improved through optimized post-treatments. To reach these objectives, a sequential order should be followed. Indeed, as schematically presented in Figure 2, the creep performances of a sample depend on the mechanisms that take place at the microscopic scale within the material. They depend on the microstructure and test conditions, i.e., loading and temperature. The formation of the microstructure, starting with the LPBF manufacturing process and potentially followed by post-treatments, is crucial for understanding the underlying creep mechanisms.

In what follows, Section 2 will lay emphasis on the microstructure of LPBF IN718 while introducing its different phases and discussing the characteristic process-induced hierarchical microstructure. The influence of common post-treatments on those microstructural features will also be described. In Section 3, general creep mechanisms within metals will be presented along with their related features and existing creep models. In Section 4, the focus will be on dominant creep mechanisms occurring within LPBF-produced IN718, based on experimental campaigns from the literature. In this section, the final properties of IN718 will be linked to the microstructure. Finally, conclusions will be drawn about the most convenient features for LPBF IN718 to sustain creep, along with the post-treatments required to obtain these optimal features. As process-induced anisotropy significantly influences mechanical behavior, the importance of sample orientation in relation to the building direction will be reiterated.

## 2. Microstructure of LPBF IN718

IN718 is a Ni-Cr-Fe FCC polycrystalline austenitic superalloy rich in Nb. Its main strengthening mechanism is precipitation hardening, which is due to the presence of nanoscale metastable γ′ and γ″ precipitates that are coherent with the γ matrix [1,2,3,6,17,18]. The mechanical performance of IN718 is highly sensitive to microstructural features [19], which has spurred extensive research into its metallurgy, manufacturing processes, and heat treatments [6]. The nominal composition of IN718, shown in Table 1, includes nickel (Ni) as the base element, with chromium (Cr), iron (Fe), niobium (Nb), and molybdenum (Mo) as primary alloying elements, and smaller amounts of titanium (Ti), aluminum (Al), and carbon (C).

The distinguishing characteristics of IN718 compared to other Ni-based superalloys are, on the one hand, the relatively high content of Nb, which promotes the formation of γ″ and δ precipitates. On the other hand, IN718 has a low content of Al and Ti, which explains the lower phase fraction of γ′ compared to other Ni-based superalloys.

### 2.1. Phases Present Within IN718 and Their Influence on Mechanical Properties

The alloy is composed of the following phases for which related impacts on mechanical performances are recalled:

- The γ matrix is a Ni-rich primary phase that is supersaturated in Cr and Fe (FCC, A1) [3,5,6]. This matrix phase is the main feature that provides both ductility and corrosion resistance to the alloy.
- $\gamma^{\prime }$ precipitates (FCC, $L1_{2}$) are structural intermetallic compounds based on the formula $Ni_{3}\left(\mathrm{Al},\mathrm{Ti},\mathrm{Nb}\right)$, which are coherent and poorly misfitting with the γ matrix [3,6,24]. The solid-state precipitation of γ′, together with γ″, enhances the strength, hardness, and creep rupture life of IN718 by inducing coherency strains, i.e., local deformations of the lattice at coherent interfaces. The influence of γ′ on the material strength is, however, less significant compared to that of γ″ due to its lower volume faction (1:3 to 1:4), reduced misfitting to the γ matrix, and lower boundary energy [3,24,25,26,27]. The precipitation of γ′ is mainly controlled by the Nb content and the thermal history of the alloy. γ′ exhibits a circular/cuboidal morphology with an average size of around 20 nm [28,29].
- γ″ precipitates (BCT, $D0_{22}$) are structural intermetallic compounds based on the formula $Ni_{3}Nb,$ which are coherent but misfitting with the γ matrix [24,30,31,32]. γ″ precipitates provide the highest strengthening effect through precipitation hardening due to their high-volume fraction, especially in Nb-rich regions [6,28,33], and due to their high coherency strains [24,28]. γ″ are reported to be metastable since they tend to transform into brittle and incoherent orthorhombic δ phases under equilibrium conditions. δ formation is, however, always preceded by the precipitation of γ″ up to 900 °C, due to the incoherence of δ with the matrix, to its sluggish precipitation kinetics, and to its much higher nucleation barrier [34,35]. Similar to γ′, the precipitation of γ″ is controlled by the Nb content and the thermal history. γ″ precipitates are shaped like ellipsoid discs with an aspect ratio of around 5, with the major axis around 30–50 nm [20]. Nevertheless, some spherical/cuboidal γ″ precipitates can grow up to 300 nm in recrystallized conditions [20,29].
- MC represents carbides, with M being Ti or Nb (B1/$E9_{3}$) [6,28,36]. Carbides can provide different effects depending on their size and composition. The most notable are related to grain growth, Grain Boundary Sliding (GBS) inhibition, and precipitation hardening [6,37]. Gao et al. [36] reported two main types of niobium carbide inclusions, which are the coarse and stable primary NbC formed during solidification and the thin secondary NbC formed at grain boundaries (GBs) during aging. The latter is responsible for oxygen-assisted crack growth as they undergo both oxidation and decomposition into brittle niobium oxides ($Nb_{2}O_{5}$-type) during high-temperature sustained loading. This could increase the crack growth rate by almost four orders of magnitude. In addition, small carbides (~200 nm) will provide strength through precipitation hardening, while coarse ones (~2 μm) will reduce ductility but improve creep performances by inhibiting GBS [6,37]. Note that nanoscale TiC particles distributed at subgrain boundaries are coherent with the matrix. Therefore, such precipitates remain stable upon high-temperature solution annealing and have been reported to improve mechanical behavior at elevated temperatures [38].
- TiN indicates stable and detrimental brittle phases that lead to local stress peaks in the alloy, significantly reducing the low-cycle fatigue life of IN718 [39,40,41]. Those nonmetallic inclusions form during the solidification stage due to the segregation of refractory Ti. The latter displays an extremely high affinity to N, which is introduced during the elaboration process. High cooling rates and large discrepancies in thermal expansion coefficients between the inclusion and matrix lead to micro-crack formation in the precipitates and to intragranular cracks after loading [42].
- δ phases (orthorhombic, $D0_{a}$) are structural intermetallic compounds based on the formula $Ni_{3}Nb$, which are stable but brittle and are incoherent with the γ matrix [6,43,44]. The precipitation of metastable γ″ up to 900 °C [34,35] triggers that of the δ phase, the former particle serving as a precursor for the latter, which exhibits sluggish precipitation kinetics. Therefore, both γ″ precipitates and δ phases share the same chemical composition. The shape of the δ phase is described as needle/plate-like. The impact of δ phases on the mechanical properties of IN718 is complex and linked to both its relative volume fraction and size. In fact, little amounts of small δ phases can be beneficial to the creep resistance of wrought IN718 by inhibiting grain growth and GBS. Conversely, coarsened δ phases deplete the alloy from Nb, which is required for γ″ precipitation. Coarse δ precipitates also lead to the formation of cracks [6,18,45,46].
- Laves phases (HCP, C14) are metastable, brittle, and bulky phases based on the formula ${\left(Ni,Fe,Cr\right)}_{2}(Nb,Mo,Ti)$ [3,47,48]. Laves phases are topologically close-packed eutectic phases that tend to form in highly Nb-segregated areas. They play an important role in decreasing mechanical performances by being favorable sites for cavity and crack nucleation. They also deplete the matrix from Nb and reduce its corrosion resistance, causing ductile fractures to occur [6,17,48,49]. Laves appear as islands and display different compositions, leading to different solvus temperatures [28]. The presence of Laves phases is one of the main reasons for the need for thermomechanical post-treatments, driving extensive research [4,26,38,45,48,50,51].

Note that all the precipitates that were mentioned above are often identified at room temperature, using either scanning or transmission electron microscopies. Such an approach is possible for cast and wrought alloys due to the size of the solidification precipitates (carbides, δ or Laves phases), which are around microns. However, these precipitates are more difficult to observe in the As-Built (AB) conditions for IN718 processed by LPBF. This can be explained either by their nanometric size or by their association with elemental segregations that occur thanks to the steep thermal gradients achieved during cooling. Therefore, the so-called “precursors” for both δ and Laves phases will be distributed within subgrain boundaries. Innovative and non-destructive Synchrotron or high-speed X-ray techniques have already been used to detect both the presence and the spatial distribution of these precipitates for cast and wrought Inconel alloys [52]. The same improved X-ray methods are now expanding to AM parts not only for the same precipitate identification purpose but also to tackle other features such as phase transformation kinetics both under AM processing and during subsequent heat treatments [16,53], internal stresses [16,54], dislocation density assessment [55], or defects and discontinuities within AM parts [16,56,57]. Nevertheless, these techniques are still limited in their ability to determine the size and/or the composition of precipitates when in situ phase transformations take place.

To summarize, on the one hand, IN718’s behavior strongly relies on the precipitation strengthening effect of intermetallic precipitates γ′ and γ″, the latter accounting for the most important effect. This is due to its higher volume fraction, boundary energy, and lattice misfit, leading to higher coherency strains [58,59]. Indeed, γ″ creates ~2.9% coherency strains within the γ matrix, while the value is around 1.25% for γ′ [60]. Note that some specific phenomena are reported in the literature such as $\gamma^{\prime }/\gamma \prime \prime$ co-precipitation, leading to complex deformation mechanisms such as spontaneous stacking fault transition, formation of superlattice intrinsic stacking fault, anti-phase boundary, and other complex stacking faults. This co-precipitation should be considered for advanced crystal plasticity models [61,62,63,64]. Precipitation strengthening within IN718 is, however, mitigated at higher temperatures due to the transformation of metastable $\gamma^{\prime \prime }$ into stable δ between 700 °C and 1000 °C [27,60,65]. Note that according to some authors, δ particles might also act as precipitation strengthening particles. IN718’s strength also comes from solid-solution hardening (alloying by substitution or interstitial atoms) through the dissolution of Mo, Mn, or C. This phenomenon induces lattice strains due to radius mismatch, resulting in lattice distortion and enhanced resistance to dislocation motion, thereby increasing material strength [6,37]. A last strengthening mechanism in wrought IN718 is characterized by GBS and grain growth, inhibiting particles such as δ, carbides, and nitrides. Although such particles are expected to reduce the alloy’s ductility, its strength should be maintained as long as excessive coarsening does not occur.

On the other hand, several mechanisms tend to decrease the strength of IN718. Indeed, coarse and brittle particles such as primary MC carbides, Laves, coarsened δ, and TiN induce micro-crack, void coalescence, and cavity formation. This damage eventually leads to the premature failure of the component. Refractory elements such as Nb or Ti have a high tendency to segregate [1], removing from the matrix components required for precipitation strengthening as well as inducing the precipitation of weakening phases, namely, NbC, coarse δ, and Laves.

### 2.2. Hierarchical Microstructure

**Figure 3:**
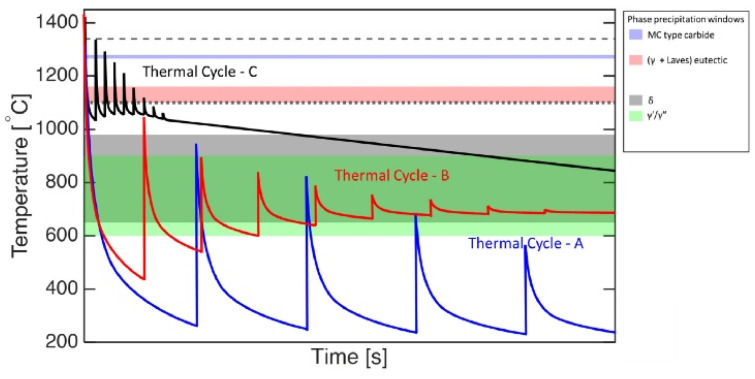
Schematic representation of the different thermal cycles in the Laser-Directed Energy Deposition (L-DED) process starting at final solidification. In Thermal Cycle–A, enough time is left between layers to avoid heat accumulation, in Thermal Cycle–B and –C, insufficient idle time is left, inducing heat accumulation. Dotted line defines the Solidus, dashed line defines the Liquidus. Reproduced from [2].

Additive manufacturing (AM), particularly Laser Powder Bed Fusion (LPBF), produces unique microstructures compared to Conventional Manufacturing (CM) techniques due to its distinct processing conditions [9]. Steep thermal gradients result from the rapid melting and cooling of the powder, while cyclic thermal loading arises from adjacent MPs and successive layers [66]. Consequently, the thermal history of a specific point in the manufactured sample can exhibit one of the trends displayed in Figure 3, depending on operating conditions. Note that remelting cycles and smaller thermal oscillations due to adjacent tracks within a layer are not depicted in this sketch. Another specificity of AM is the intrinsically directional thermal gradients, promoting epitaxial growth, globally along the sample’s Build Direction (BD). This leads to largely anisotropic microscopic features and mechanical responses [8,22,67].

**Figure 4:**
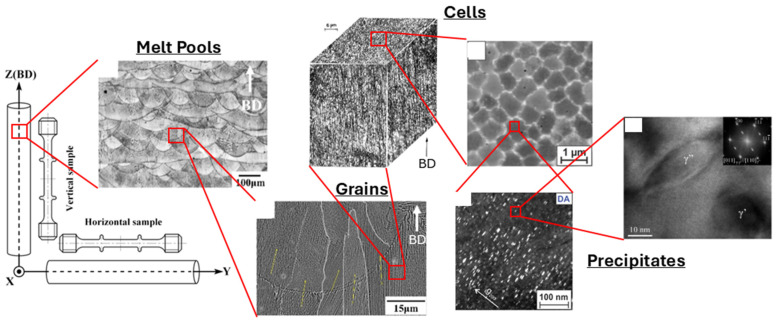
Hierarchical microstructure of AB and Direct-Aged LPBF IN718 from the sample level to nanoscale precipitates. Melt Pools, grains, and cells are identified. Pictures adapted from [8,15,18,20,69].

Due to those unusual manufacturing conditions, AB samples display what is referred to as a hierarchical microstructure with distinct features at different scales, as shown in Figure 4 [51]. First, one can observe Melt Pools of approximately 100 µm width and then elongated grains covering several MPs at the same scale [23,50]. At the microscale, dendritic/cellular substructures appear due to rapid solidification, and, at the nanoscale, strengthening precipitates are observed within the cells [68].

#### 2.2.1. Melt Pool Scale

**Figure 5:**
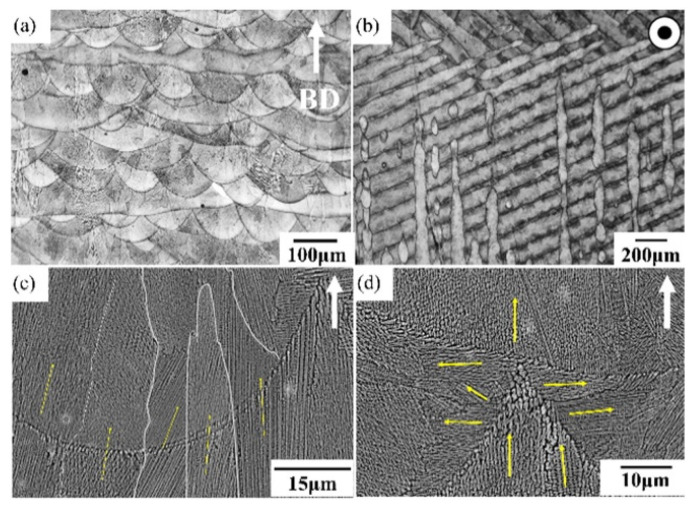
Melt Pool Boundary (MPB) microstructures of the AB specimen: (**a**) side view and (**b**) top view. The BD is shown by the white arrow. Side views of the Scanning Electron Microscope (SEM) microstructure showing (**c**) layer–layer MPBs and (**d**) track–track MPBs. The dendrite growth directions are shown by yellow arrows. Reproduced from [15].

Melt Pools, sometimes called Molten Pools, are vestiges of AM laser tracks, i.e., each MP is generated by one scan of the laser. Note that for each track within a multi-layer process, the laser partly remelts the previous layer, increasing the depth of the resulting MP. As displayed in Figure 5a, MPs are U-shaped, and each solid–liquid interface acts as a solidification front. This leads to thermal gradients normal to the MP boundary and texture generation accordingly (see dendritic growth in Figure 5d). MP dimensions (width and depth) depend on the processing parameters (mainly the laser power and the scanning speed). According to the work of Scime et al. [70] and similar to [66], for a laser power ranging from 100 W to 370 W and a scanning speed of 200 mm/s to 1400 mm/s, the MP width and depth of LPBF IN718 range from 100 µm to 200 µm and from 50 µm to 300 µm, respectively. Other parameters such as the powder layer thickness, the hatch spacing, or the scanning pattern influence the MP properties [67,71]. MP boundaries have been reported to largely impact the fracture mechanisms of additively manufactured samples for specific materials such as AlSi10Mg [72,73] due to their heterogeneous microstructural features. Therefore, extensive research is ongoing to model LPBF at the MP level to predict its shape and the thermal history of AB samples [72,74,75,76].

MPs in LPBF Inconel 718 disappear after most of the commonly used heat treatments involving at least solutioning [4,51]. Since grains rarely coincide with Melt Pool Boundaries (MPBs) due to epitaxial growth, MPBs do not seem to directly influence creep fracture mechanisms. Therefore, the impact of MPs on creep has not been extensively discussed in the literature [77]. However, remelting due to overlapping consecutive scans generates differences in grain size and dendritic growth at the track–track interfaces [67,68], which might influence the fracture behavior, as proposed by several authors [15,67,78]. This last feature emphasizes the need for an optimal scan strategy within each layer. This strategy directly defines the overlapping width.

#### 2.2.2. Grain Scale

Grains are regions of the material with a common lattice orientation that are separated from each other by Grain Boundaries (GBs). GBs are preferential sites for precipitates and voids to nucleate during mechanical testing, and they can be characterized through their misorientation angle, i.e., the lattice orientation mismatch between two adjacent crystals. According to Chauvet et al. [79], High-Angle Grain Boundaries (HAGBs > 15°) are much more sensitive to crack propagation than Low-Angle Grain Boundaries (LAGBs < 15°).

**Figure 6:**
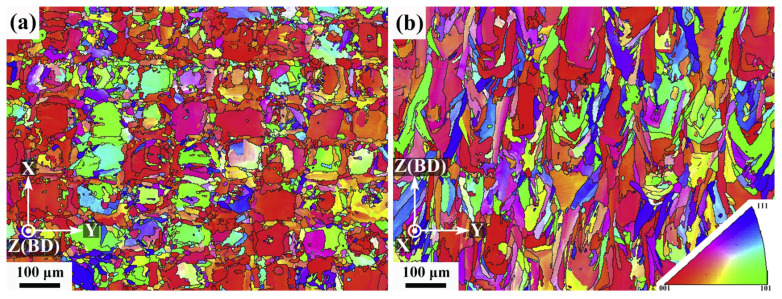
EBSD orientation maps of the sample (**a**) perpendicular and (**b**) parallel to the BD. Reproduced from [8].

For AM IN718, thermal gradients are so large that solidification occurs as cellular growth normal to the liquid–solid interface defined by the MPBs. Each group of parallel columnar cells forms a grain. In addition, partial remelting of the previous layer promotes epitaxial growth across MPs, leading to columnar grain development across layers, as displayed in Figure 5c [80,81]. According to the experimental results of Calandri et al. [81], grains for LPBF IN718 measure on average 30 µm in height but can grow up to 180 µm. Note that those dimensions largely depend on the process parameters [82]. Along with a strong <100> crystallographic texture, subgranular domains with LAGBs are observed [81]. As mentioned in the previous subsection, the thermal history in the track–track interface between two MPs is more complicated due to remelting and to non-vertical thermal gradients. This leads to small equiaxed grains with coarse cells and erratic orientation, as indicated by yellow arrows in Figure 5d [67,68]. Electron BackScatter Diffraction (EBSD) maps highlighting the peculiar grain structure normal and parallel to the BD are shown, respectively. in Figure 6a,b. Another way to characterize this process-induced anisotropy is through the average aspect ratio of the grains, defined as the length of a grain along the BD divided by its width. Calandri et al. [81] found values of 5.4, while Wu et al. [17] measured them to be around 3.37 after Solution Heat Treatment (SHT).

#### 2.2.3. Cellular Scale

**Figure 7:**
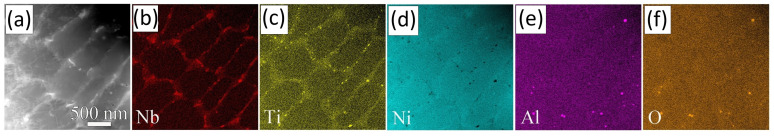
(**a**) High-Angle Annular Dark-Field Imaging in a Scanning Transmission Electron Microscopy (HAADF-STEM) micrograph of AB IN718 and (**b**–**f**) corresponding Scanning Transmission Electron Microscopy–Energy Dispersive X-ray (STEM-EDX) maps highlighting segregated elements at cell boundaries. Partial figure reproduced from [60].

As mentioned previously, the very high temperature gradient and fast cooling rate characterizing AM processes and, specifically, LPBF (~10^6^ K/s against ~3.75 K/s for casting [82,83]) lead to cellular dendritic solidification [18,68,81]. Indeed, the shape of the solidification front, also called the solidification interface, is controlled by the G/R ratio, with G being the thermal gradient and R the solidification interface velocity. A planar solidification front will be obtained for high G/R ratios (~7000 K s mm^−2^ [84]), whereas the solidification will change to a cellular, cellular dendritic, columnar dendritic, or equiaxed dendritic type, with decreasing G/R ratios. For instance, Wei et al. [84] computed a ratio between 20 K s mm^−2^ and 100 K s mm^−2^ for laser-based AM of IN718, indicating cellular or dendritic solidification structures. This coincides with experimental observations from the literature. Cellular growth is directly related to the element partition coefficient *k* (i.e., the ratio of element solubility in liquid and solid phases), which, if lower than 1, leads to segregation. In LPBF IN718, Nb, Ti, Mo, and Al segregate at the cellular boundaries due to their partition coefficients, equal to 0.28, 0.41, 0.73, and 0.79, respectively [81]. As displayed in Figure 7b,c, this phenomenon was experimentally observed by Gallmeyer et al. [60] for both Nb and Ti, the elements with the lowest partition coefficients, i.e., the largest tendency to segregate.

**Figure 8:**
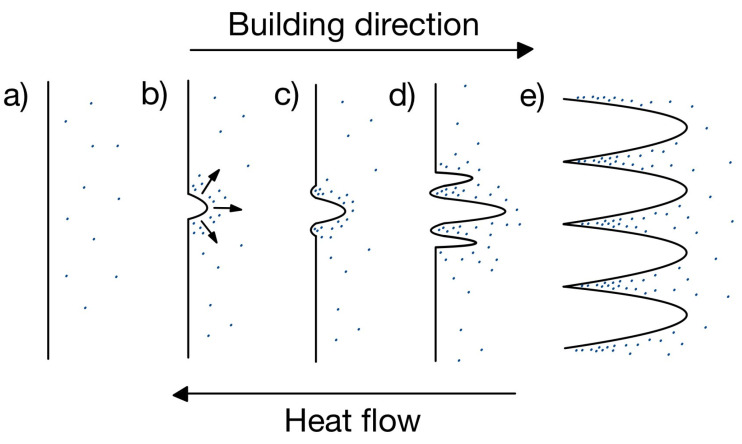
Schematic representation of cellular growth: (**a**) initial planar interface, (**b**) formation of a first protrusion, (**c**) formation of recesses, (**d**) formation of other protrusions, and (**e**) formation of cells. Blue dots are segregating elements. Inspired by [68,85].

**Figure 9:**
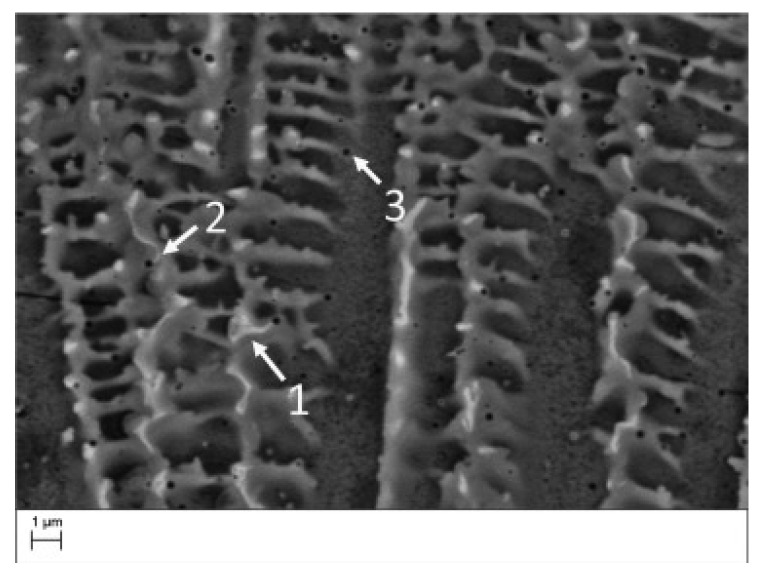
Interdendritic microsegregation inside γ-phase grains; the arrows indicate the following: 1—colonies of eutectic mixture (γ + Laves type phase); 2—divorced eutectics; 3—MC carbides (black). BSD/SEM. Reproduced from [86].

Cellular solidification is a well-known process that can be linked to element segregation, as highlighted by Porter et al. [85]. Figure 8 schematically represents the process of cellular growth. Initially, elements are distributed in the liquid ahead of the planar interface (a). A gradual reduction in temperature below the critical value leads to the formation of a first protrusion, causing elements to be rejected due to their low solubility and explaining their elevated concentration at its roots (b). Excess solute at the roots reduces the equilibrium solidification temperature, inducing the formation of recesses (c), itself causing the formation of other protrusions (d). Finally, protrusions grow along the BD (opposite to the heat flow) (e). Note that each cell displays the same lattice orientation, forming a single grain [85]. The extreme speed of the phenomenon does not allow for secondary arms to form and explains the final cellular structure. As mentioned in [18,86,87,88] and illustrated in Figure 9, the segregated solute at the roots of the cells solidifies at the lowest temperature and forms the eutectic precursor of Laves (214 ± 62 nm [60]) and carbides (25–50 nm [81]).

The level of segregation, the matrix concentration, and the dimensions of the cells depend on the process parameters. For instance, an increased solubility of Nb in the γ matrix has been reported for high cooling rates [89]. For a laser power of 175 W, a scanning speed of 620 mm/s and a hatch distance of 0.12 mm, the median diameter of the cellular substructure has been observed to measure 0.5–0.65 µm [18], between 0.5 µm and 1 µm [20] or 0.62 ± 0.18 µm [60]. The corresponding Nb enrichment in the cell boundaries has been measured to be 19.4 ± 2.4 wt% [18]. In addition, high dislocation density (1.6 ± 0.8 × 10^14^ m^−2^ [60]) due to residual stresses and high thermal gradients have been observed along the substructure boundaries [4,18].

#### 2.2.4. Subcellular Scale

**Figure 10:**
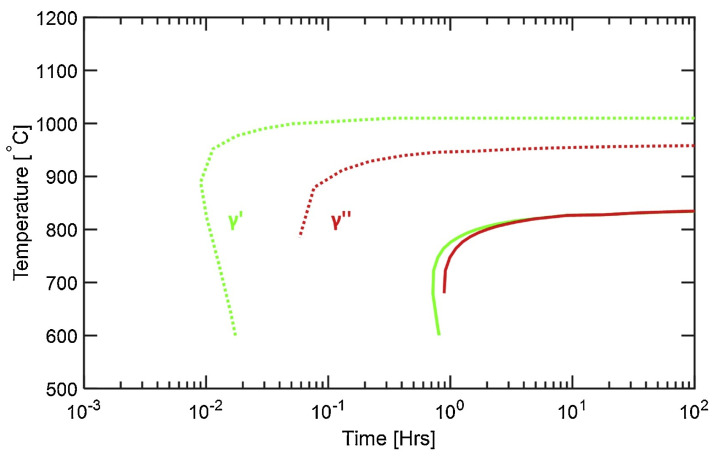
Continuous Cooling Transformation (CCT) diagram created using segregated compositions predicted from MICRESS. Dotted lines define 0.5% precipitation close to Laves phases and solid lines define 0.5% precipitation in the dendrite core. Reproduced from [90].

At the subcellular scale, one can mostly observe $\gamma^{\prime }$ and $\gamma^{\prime \prime }$ strengthening precipitates in addition to small-sized carbides. γ″ tend to precipitate as <200> textured columnar stacks with precipitates measuring 100 nm along their major axis and 25 nm along their minor axis, according to Amato et al. [20]. Spherical Nb carbides form in Nb-enriched areas, i.e., in the interdendritic regions [86]. In the AB condition, precipitation of $\gamma^{\prime }$ and γ″ is, however, not very substantial: the formation of those phases relies on solid-state phase transformations (see Figure 3 for precipitation windows) but rapid solidification does not allow for extensive precipitation, relying on diffusion, as represented in the Continuous Cooling Transformation (CCT) diagram in Figure 10. Note that an ideal size of ~23 nm for γ″ precipitates was experimentally set by Chaturvedi [13] in order to reach the highest level of strengthening in creep without forming incoherent interfaces. Due to Nb and Ti segregation, a gradient of precipitate density is observed between the cell boundary and the cell core, leading to a corresponding gradient in mechanical properties [2]. Solution or homogenization heat treatments are often recommended to solve the problem of insufficient precipitation of strengthening phases due to the inhomogeneous distribution of Nb and Ti.

### 2.3. Heat Treatments

The out-of-equilibrium microstructure of AB LPBF IN718 requires post-treatments to achieve stable and enhanced mechanical properties. Certain out-of-equilibrium features, such as matrix supersaturation that improves solid-solution strengthening, are advantageous for mechanical behavior; however, the AB microstructure exhibits lower creep properties than CM alloys [12,15,67,91,92]. Table 2 compares creep features of IN718 obtained from both elaboration routes. CM samples outperform AM ones by approximately one order of magnitude. Note the abnormally low performances of wrought IN718 in Gao et al. [92], which is probably due to a different heat treatment. Additional data are available for compression [18] and Small Punch Creep [78], but the results are different due to the different mechanisms taking place.

An additional clue indicating the need for post-treatments was highlighted by Pröbstle et al. [18]: the AB microstructure is very unstable regarding its phases. In their compressive tests at 630 °C, a rapid softening due to phase transformations was observed. According to Sundararaman et al. [44], despite the relatively low temperature, metastable δ tend to nucleate at γ″ stacking faults, assisted by the high applied stress (900 MPa). This phenomenon is still observed after AMS 5662 standard heat treatment (see Table 3) [67]. This instability is due to the highly segregated Nb at the cellular boundaries, leading to enhanced precipitation in these areas. In addition, instabilities in the microstructure also appear through grain growth during thermal exposure [19].

Initially, researchers relied on conventional heat treatments developed for cast and wrought IN718 following AMS 2774, AMS 5383, AMS 5662, AMS 5663, and AMS 5664 standards [94] to post-process AB LPBF IN718 (see Table 3 for references). Table 3 gathers all heat treatments associated with LPBF IN718 creep tests found in our literature review. The AMS 5662 standard is by far the most used post-treatment. It will be considered as the reference heat treatment for the next sections. Numerous studies have concluded that standard heat treatments are unsuitable for LPBF IN718 due to significant differences in the initial microstructures of CM and AM samples. Zhou et al. [95] concluded that the 980 °C solution annealing pertaining to AMS 5662 neither permits the dissolution of detrimental Laves phases nor decreases element segregation. Conversely, precipitation of detrimental δ phases occurs. Similar results were found within numerous works [3,4,68,88,96]. AMS 5662 HT samples generally show poorer results than CM alloys [17,91,97,98]. Sanchez et al. [67] and Gao et al. [92] were, however, able to obtain better creep performances for LPBF AMS 5662 IN718 than for wrought IN718. In the research by Sanchez et al., an increase of 24% in the creep life of LPBF IN718 compared to wrought IN718 was observed, but minimum creep rates were similar, and the elongation to fracture was almost four times larger in the wrought alloy. Regarding Gao et al., only the creep life was compared, and the better performance of the AM sample was probably due to the abnormally low creep life of the wrought sample, as previously mentioned. Note that Sanchez et al. [67] still recommended the development of a more appropriate heat treatment, which could improve the creep resistance even further [19].

To summarize, there is a clear need for heat treatments on AB LPBF creep samples, and the conventional treatments developed for cast and wrought alloys are not an acceptable option. Indeed, despite some authors finding acceptable performances for LPBF IN718 + AMS 5662, it is largely recognized that results can be significantly improved with dedicated post-treatments.

Table 3, comparing the different post-treatments applied to improve the creep properties of LPBF IN718, is classified into four main categories that will be developed later: Hot Isostatic Pressing (green), homogenization (grey), solution (yellow), and double-aging (blue). Since the terms homogenization and solution are often used interchangeably in the literature, this study uses them based on their microstructural implications in LPBF IN718. Note that Table 5 in Section 4.4 describes the microstructural features associated with some of these post-treatments.

**Table 3:** Comparison of post-treatments used in the literature on LPBF IN718 for creep testing. The heat treatment (HT) number is used to refer to a specific line.

HT	References	Hot Isostatic Pressing	Homogenization	Solution	Aging	Standard
1	[99]	1120 °C for 4 h at 100 MPa	(Before HIP) 1065 °C for 1.5 h	1025 °C for 0.5 h	850 °C for 0.5 h + 650 °C for 16 h	/
2	[99]	1120 °C for 4 h at 100 MPa	(Before HIP) 1065 °C for 1.5 h	960 °C for 1 h	720 °C for 3 h + 620 °C for 5 h	/
3	[99]	1120 °C for 4 h at 100 MPa	(Before HIP) 930 °C for 1 h	960 °C for 1 h	720 °C for 8 h + 620 °C for 10 h	/
4	[100]	1163 °C for 4 h at 100 MPa	/	1066 °C for 1 h	718 °C for 8 h + 621 °C for 8 h	/
5	[101]	1163 °C for 3 h at 103 MPa	/	954 °C for 1 h	718 °C for 8 h + 621 °C for 8 h	AMS 5663 + AMS 2774
6	[99]	1120 °C for 4 h at 100 MPa	/	1020 °C for 1 h	850 °C for 0.5 h + 650 °C for 16 h	/
7	[96]	1180 °C for 4 h at 175 MPa	/	/	720 °C for 8 h + 620 °C for 10 h	/
8	[102]	1175 °C for 4 h at 100 MPa	/	/	718 °C for 8 h + 621 °C for 8 h	/
9	[7]	1160 °C for 4 h at 150 MPa	/	/	710 °C for 8 h at 100 MPa + 610 °C for 8 h at 90 MPa	/
10	[103]	1200 °C for 4 h at 103 MPa	/	/	/	/
11	[96]	1180 °C for 4 h at 175 MPa	/	/	/	/
12	[104]	Homogenization	1100 °C for 1 h	980 °C for 1 h	720 °C for 8 h + 620 °C for 8 h	/
13	[103,105]		1093 °C for 1–2 h	954–982 °C for >1 h	718 °C for 8 h + 620 °C for 10 h	AMS 5383
14	[106]		1065 °C for 1.5 h	980 °C for 1 h	760 °C for 10 h + 650 °C for 8 h	/
15	[1,106,107]		1065 °C for 1.5 h	/	760 °C for 10 h + 650 °C for 8 h	/
16	[17]		/	1180 °C for 12 h	720 °C for 8 h + 620 °C for 8 h	/
17	[96]		/	1180 °C for 4 h	720 °C for 8h + 620 °C for 10h	/
18	[96]		/	1180 °C for 1 h	720 °C for 8 h + 620 °C for 10 h	/
19	[108]		/	1130 °C for 1 h	720 °C for 8 h + 620 °C for 8 h	/
20	[96]		/	1120 °C for 1 h	720 °C for 8 h + 620 °C for 10 h	/
21	[17]		/	1080 °C for 12 h	720 °C for 8 h + 620 °C for 8 h	/
22	[92]		/	1080 °C for 1.5 h + 980 °C for 1 h	720 °C for 8 h + 620 °C for 8 h	/
23	[92]		/	1080 °C for 1.5 h	720 °C for 8 h + 620 °C for 8 h	/
24	[17]		/	1080 °C for 1 h	720 °C for 8 h + 620 °C for 8 h	/
25	[91]		/	1065 °C for 1 h	760 °C for 10 h + 650 °C for 8 h	/
26	[96]		/	1065 °C for 1 h	720 °C for 8 h + 620 °C for 10 h	/
27	[109]		/	1060 °C for 1 h	760 °C for 10 h + 650 °C for 8 h	AMS 5664
28	[108]		/	1060 °C for 1 h	720 °C for 8 h + 620 °C for 8 h	/
29	[96]	Solution		1045 °C for 1 h	720 °C for 8 h + 620 °C for 10 h	/
30	[18]			1000 °C for 1 h	720 °C for 8 h + 620 °C for 8 h	/
31	[1,106]			980 °C for 1 h	760 °C for 10 h + 650 °C for 8 h	/
32	[96]			980 °C for 1 h	720 °C for 8 h + 620 °C for 10 h	/
33	[7,17,67,91,92,97,98,104,108]			980 °C for 1 h	720 °C for 8 h + 620 °C for 8 h	AMS 5662
34	[15]			980 °C for 1 h	718 °C for 8 h + 621 °C for 10 h	/
35	[110]			960 °C for 1 h	720 °C for 8 h + 620 °C for 8 h	/
36	[18]			930 °C for 1 h	720 °C for 8 h + 620 °C for 8 h	/
37	[111]			850 °C for 2 h	720 °C for 8 h + 621 °C for 8 h	/
38	[18,98]	Double Aging			720 °C for 8 h + 620 °C for 8 h	/
39	[15]				718 °C for 8 h + 621 °C for 10 h	/

#### 2.3.1. Hot Isostatic Pressing (HIP)

Usually, HIP is used to relieve stresses and to reduce process-induced porosities [7,99,100,101,102,112], which play a major role in reducing creep performances. HIP generally leads to recrystallization and grain growth due to the high temperature associated with pressure. Indeed, among the seven reported studies, six of them explicitly reported recrystallization [7,96,99,100,101,103] with the presence of annealing twins [96,99,100]. Note that McLouth et al. [101] observed that with a defocused laser, elongated grains, and texture can be preserved through HIP and solution + double-aging treatment. Carbide precipitation and coarsening within the grains and at GBs are observed due to lower solidification rates during HIP [96]. Kaletsch et al. [7] also observed that carbides formed during the LPBF process that were not dissolved during HIP, appearing at the GBs of the AB microstructure. In addition to these recrystallization and grain growth effects, HIP at high temperature can dissolve both Laves and δ phases and fully homogenize Nb in the γ matrix [7].

#### 2.3.2. Homogenization and Solution Annealing

Homogenization and solution annealing both aim at reducing element segregation and dissolving detrimental phases such as Laves or δ [3,7,18,67] while modifying the grain structure in the case of high-temperature processes [12,17,99]. Sadek et al. [99] observed that an increase in the soaking time of solution annealing (SA) promotes the precipitation and growth of coarser strengthening phases, probably due to a better redistribution of Nb. They also observed serrated grain boundaries for a so-called “stress relief” at 930 °C, which were also reported by Yeh et al. [113] and Kuo et al. [96].

As mentioned above, the regular 980 °C SA from the AMS 5662 standard does not lead to the homogenization of segregated elements and does not dissolve detrimental Laves. The treatment is applied within the range of δ phase stability. This leads to its extensive precipitation within GBs and interdendritic areas due to a high concentration of Nb [96,108]. In addition, δ phases provide a pinning effect, preventing grain growth and recrystallization [96].

Increasing the homogenization/solution temperature reduces the precipitation of interdendritic δ by homogenizing Nb and Ti until a critical value that fully prevents δ phase precipitation. This treatment allows for partial or full recrystallization and promotes grain growth. Driving forces for recrystallization and grain growth are attributed to both temperature and residual stresses [114]. Uneven distribution of residual stresses might induce anisotropic grain growth [96]. The transition temperature that triggers recrystallization and grain growth is directly linked to the presence of δ, for which different solvus temperatures have been reported: ~1000 °C for Azadian et al. [35], ~1010 °C for Shi et al. [1] and Pröbstle et al. [18], and 1045 °C for Kuo et al. [96]. From the literature, it seems that a solution/homogenization treatment above 1060 °C does not lead to the precipitation of the δ phase [1,17,92,96,108,109], unless it is followed by a lower temperature solution/homogenization step [92,103,104]. However, note that a high aging temperature might lead to precipitation of δ even if the solution/homogenization step was expected not to lead to δ precipitation [106,107,109]. Carbides are observed along GBs and coarsen above 1120 °C [96]. Pröbstle et al. [18] and Zhang et al. [4] indicated that temperatures above 1050 °C lead to recrystallization and loss of substructures.

#### 2.3.3. Aging

Aging is used to enhance the precipitation of the γ′ and γ″ strengthening phases [18,110] and largely relies on the efficiency of Nb and Ti homogenization during previous heat treatments. It has, however, been reported that an increased soaking time during aging leads to an increased precipitation of GB precipitates and a decreased grain size. It also promotes the precipitation of intragranular and intergranular carbides [99]. As explained above, several authors have observed the precipitation of δ phases due to high aging temperatures, while the solution/homogenization temperature should not lead to their precipitation [106,107,109]. However, a direct aging treatment at moderate temperatures (~720 °C and ~620 °C) leads to γ′/γ″ precipitation but does not relieve stress and cannot dissolve interdendritic Laves phases [15,18,98]. Note that Kuo et al. [15] observed smaller particle-shaped δ phases resulting from the coherency loss between the γ matrix and γ″ precipitates, due to coarsening of the latter.

#### 2.3.4. Heat Treatments Summary

**Figure 11:**
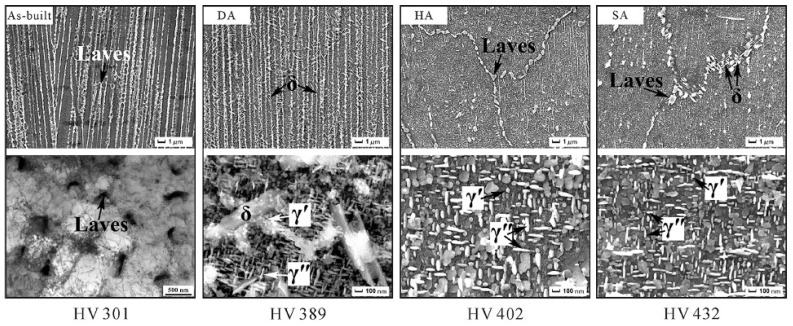
SEM micrographs of AB, DA, HA, and SA samples at the micro- and nanoscales with the associated hardness values. Reproduced from [68].

**Figure 12:**
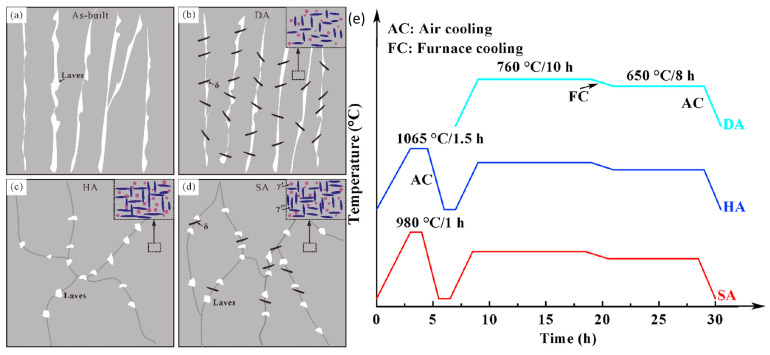
Schematic illustrations of the microstructure of the (**a**) AB sample and after (**b**) DA, (**c**) HA, and (**d**) SA heat treatments. The insets in the right corner of (**b**–**d**) illustrate the γ′ and γ″ phases in the γ matrix. (**e**) Heat treatment routes: DA, HA, and SA. Reproduced from [68].

Overall, heat treatments of IN718 can be decomposed into three main ranges that will be hereafter referred to as homogenization, solution, and aging. This convention is not consistent with all references but is used for clarity. The first one (homogenization) is a high-temperature homogenization above ~1060 °C that leads to partial homogenization of Nb and Ti, does not contribute to the precipitation of the δ phase, and allows for at least partial recrystallization and grain growth. The second one (solution) is a medium-temperature solution annealing between ~850 °C and ~1060 °C that does not homogenize enough Nb and Ti and contributes to δ precipitation, usually inhibiting recrystallization and grain growth. Finally, the third range of heat treatments (aging) corresponds to processes between ~620 °C and ~760 °C that promote $\gamma^{\prime }/\gamma \prime \prime$ precipitation but contribute to δ apparition if the soaking time or the temperature is too high. The complete post-treatment usually consists of combinations of those processes such as homogenization–solution–aging (HSA) [92,103,104,105,106], homogenization–aging (HA) [1,17,91,92,96,106,107,108,109], solution–aging (SA) [1,7,15,17,18,67,91,92,96,97,98,104,106,108,110,111], and direct- or double-aging (DA) [15,18,98]. Figure 11 and Figure 12, respectively, show micrographs and schematics of AB, DA, HA, and SA microstructures. The DA sample retains the dendritic microstructure with the addition of small γ′/γ″ precipitates. A large amount of δ precipitates is also observed due to a lack of Nb and Ti homogenization and due to the high aging temperature (760 °C). The HA sample does not retain the dendritic microstructure, and little to no δ is observed. Laves remain at GBs, and γ′/γ″ phases are coarser and in higher quantity than for the SA and DA treatments. δ phases are present at GBs in addition to Laves after the SA treatment. γ′/γ″ phases after the SA treatment seem slightly smaller than in the HA sample. An HIP stage can be added on top of those treatments or replace the homogenization stage to reduce material porosity at the cost of increased energy consumption.

These conclusions on microstructural features associated with HA, SA, and DA are summarized in Table 6 in Section 4.4, with the relative efficiency of each heat treatment for creep behavior enhancement.

## 3. Creep in Metals

At elevated temperatures, materials undergo a time-dependent mechanism referred to as creep. In fact, the general rule states that metals exhibit creep behavior above a threshold temperature equivalent to 0.3–0.35 of their melting temperature (T_m_) [115,116,117]. Creep is a diffusion-driven process that leads to premature, permanent deformation at loadings inferior to the conventional elastic limit of the material. This deformation is slow and continuous, eventually causing fracture of the component. Diffusion, characterized by the relative motion of atoms throughout the crystal lattice, is a required mechanism for creep. Mathematically, elastic and plastic deformations are functions of stress and temperature:

(1) $$\varepsilon_{elastic/plastic}=f(\sigma ,\;T)$$

while creep deformation is also time-dependent:

(2) $$\varepsilon_{creep}=f(\sigma ,\;T,\;t)$$

**Figure 13:**
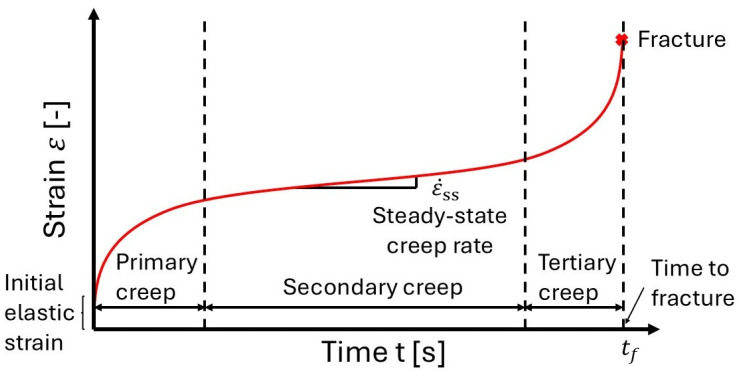
Usual strain–time creep curve.

Typically, the creep deformation of a material is modeled through its strain–time curve. The latter usually consists of three regions as depicted in Figure 13: the primary creep, the steady-state region (secondary creep), and the damage zone (tertiary creep) until fracture.

### 3.1. Creep Mechanisms

Creep is mostly divided into two mechanisms: diffusional creep and dislocation creep, also known as Power-Law Creep (PLC). Note that dislocation creep is also a diffusion-assisted process. These mechanisms manifest at varying levels of temperature and stress and are commonly illustrated in creep mechanism maps, such as the one shown in Figure 17a. At high temperatures and stresses, dislocation creep dominates, whereas lower stress levels favor diffusional creep. For temperatures below the 0.3–0.35 T_m_ threshold and low stress values, a simple elastic response is observed. For stress values above the yield strength of the material, a plastic response is observed. Some authors consider Grain Boundary Sliding (GBS) as an additional creep mechanism [14,118,119], but most of them regard it as a direct consequence of the two main mechanisms [115,116,120]. Creep fracture is also diffusion-driven, occurring through void nucleation and growth due to diffusional flow and stress accumulation, primarily at GBs and incoherent precipitate interfaces.

#### 3.1.1. Diffusional Creep

**Figure 14:**
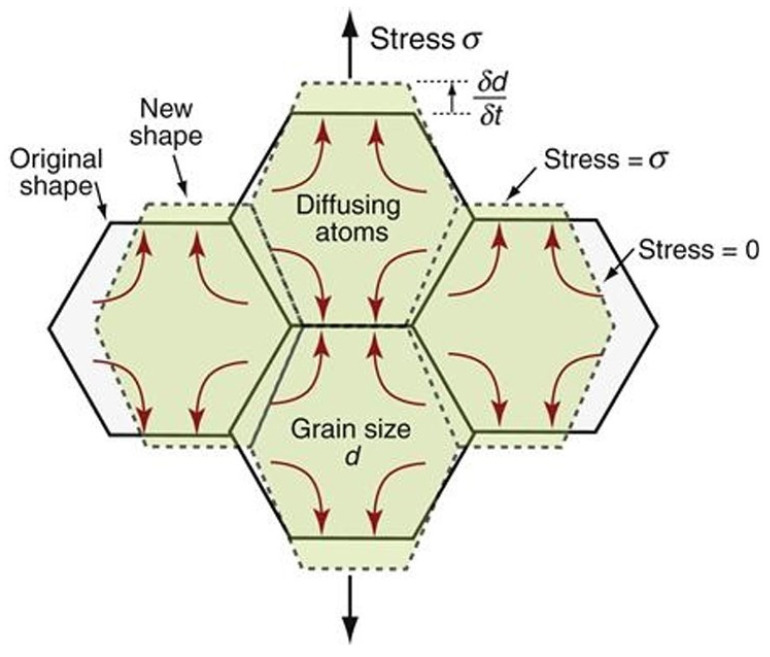
Schematic representation of Nabarro–Herring diffusional creep. Reproduced from [116].

For polycrystalline materials, diffusional creep takes place at low stress and occurs through the diffusion of atoms. Diffusion is a stress-driven process that is enabled by relatively high temperatures. Atoms migrate from GBs parallel to the loading direction to GBs perpendicular to it. At low temperatures, atoms diffuse along GBs, a mechanism known as Coble creep, while at high temperatures, diffusion occurs through the bulk, referred to as Nabarro–Herring creep [120]. This motion of atoms under constant stress deforms the polycrystalline structure, elongating the grains along the loading direction, as depicted in Figure 14.

Diffusional creep can be modeled through a simple constitutive equation of the steady-state creep rate [115,116]:

(3) $${\dot{\varepsilon }}_{ss}=A\frac{e^{-Q/RT}}{d^{2}}\sigma$$

where A is a material parameter, Q is the activation energy for creep, R is the universal gas constant, T is the temperature, d is the grain size, and σ is the applied stress. A form of Fick’s law can be recognized, where diffusion is driven by stress rather than a concentration gradient, which explains the linear dependence on the applied stress. The steady-state creep rate is also inversely proportional to the square of the grain size because, for larger grains, atoms must diffuse across a longer distance.

GBS occurs as a secondary process to accommodate relative motion between adjacent grains, preventing void nucleation at GBs, and GB separation [115,120].

#### 3.1.2. Dislocation Creep

**Figure 15:**
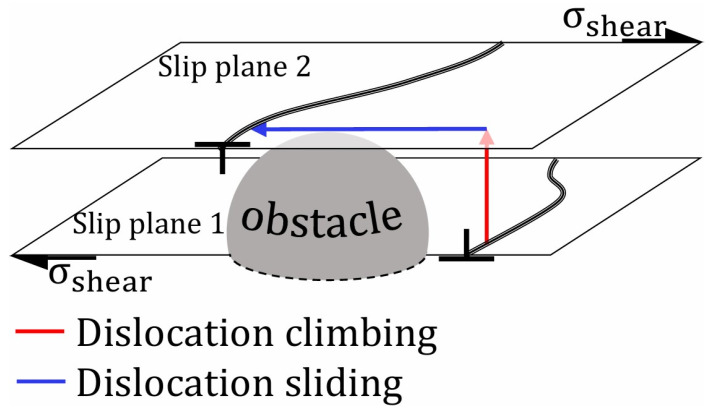
Schematic representation of dislocation creep. Reproduced from [122].

Regular plastic deformation is due to the motion of dislocations as they glide through the crystal lattice. Dislocation motion is hindered by intrinsic lattice resistance, solute atoms, precipitates, grain boundaries, or other dislocations. During dislocation creep, dislocations are unlocked by atom diffusion, allowing them to climb over obstacles, as depicted in Figure 15. Similar to diffusional creep, atom diffusion during dislocation creep is driven by the applied stress and the temperature. Since diffusion is required for the climb process, dislocation creep occurs above 0.3–0.35 T_m_ [115,116]. Up to 0.5 T_m_, core diffusion (motion of atoms along the dislocation core, also known as pipe diffusion [121]) is dominant, while between 0.5 and 0.99 T_m_, bulk diffusion (motion of atoms from the lattice to the dislocation line, also known as lattice diffusion [121]) is the main process. Once unlocked through climbing, dislocations glide (slide) until encountering a new obstacle, restarting the cycle. The subsequent glide–climb sequences explain the progressive and continuous features of creep [115,116].

Due to its dependence on diffusion, dislocation creep can be modeled similarly to diffusional creep through its steady-state creep rate [115,116]:

(4) $${\dot{\varepsilon }}_{ss}=Ae^{-Q/RT}\sigma^{n}$$

where A is a material parameter and n is the stress exponent, usually comprised between 3 and 8 [115]. According to Ashby et al. [116], this exponential dependance on stress is difficult to explain and partly comes from the non-constant density of dislocations.

### 3.2. Creep Fracture

**Figure 16:**
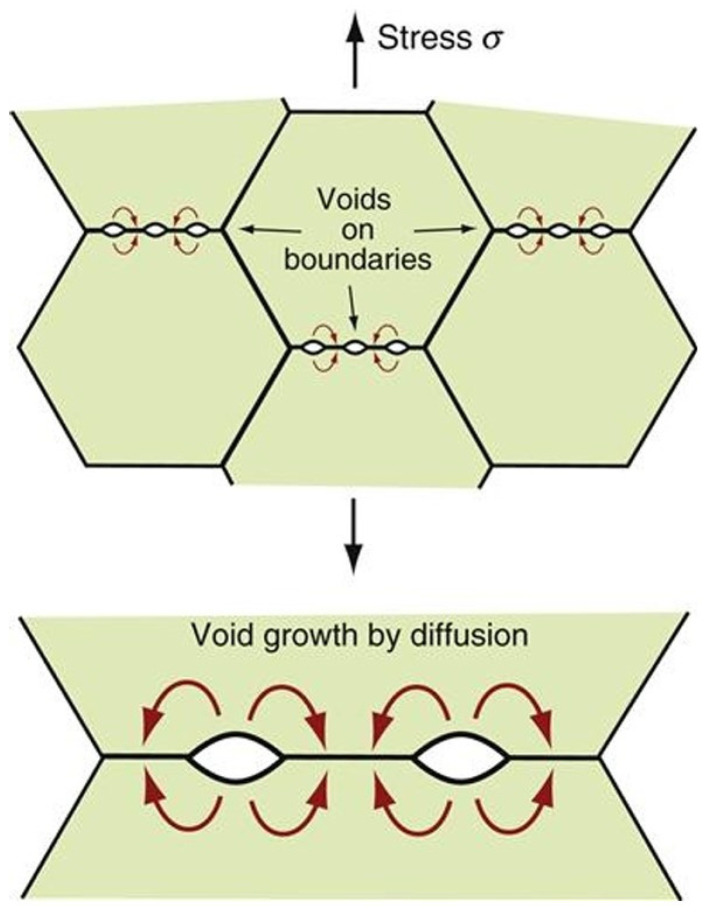
The growth of voids on grain boundaries by diffusion. Reproduced from [116].

Similar to creep deformation, creep fracture depends on both diffusion and dislocation motion. Indeed, cavities nucleate along GBs perpendicular to the applied stress due to atom migration. These cavities then grow into voids that cannot sustain loads, and stress accumulates along intact GBs, which continuously diminish in size as voids grow, as shown in Figure 16 [115,116].

GBs are weak points because they are full of impurities, defects, segregated elements, and secondary phases. Those features, in addition to the misorientation characterizing a GB (minimum 15°), lead to dislocation piling-up and local peaks of stress promoting interface decohesion. These characteristics indicate that typical creep fracture is intergranular. The coalescence and growth of cavities and voids along perpendicular GBs lead to the formation of cracks, explaining the fast deformation until fracture during the tertiary creep regime. However, transgranular fractures could also be observed in the case of strong GBs (displaying a low number of defects) with incoherent precipitates within the matrix [123], potentially along substructures (see Section 2).

Note that in addition to cavities (also known as r-type voids), wedge-type cracks (sometimes referred to as w-type cracks) also play an important role in creep damage [123]. They often appear at triple points as a consequence of either GBS or void accumulation [17].

### 3.3. Creep Mechanism Identification and Diagrams

For a long time, material scientists identified creep mechanisms (either diffusional or dislocation-based) through the stress exponent n. It is unitary in the case of purely diffusional creep (since it directly comes from stress-assisted diffusion through Fick’s diffusion formula [115,116]) and between 3 and 8 for dislocation-based creep. However, this approach was recently questioned by Sandström [124], who argued that stress exponents ranging from 1 to 50 have been obtained in the literature for dislocation creep, making this mechanism identification method obsolete. However, despite Sandström’s recommendations, this approach is still largely used in the literature; hence, it will be considered in this paper.

**Figure 17:**
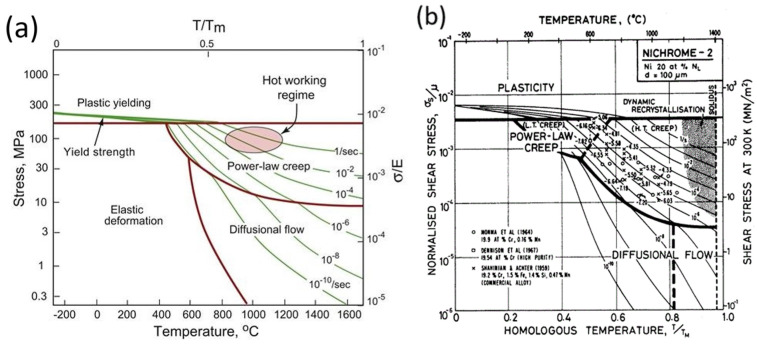
(**a**) Deformation mechanisms at different stresses and temperatures of a nickel alloy. Reproduced from [116]. (**b**) Nickel-20 at.% chromium with a grain size of 100 µm. The temperature is normalized by the melting point of pure nickel (1726 K). Reproduced from [125].

No creep mechanism map has been explicitly developed for LPBF IN718, but the closest one that is available is for NICHROME alloys with 20% Cr, as depicted in Figure 17b. Ideally, these maps should be created for each material and updated with changing microstructural features.

### 3.4. Creep Modeling

While a comprehensive discussion of all existing methods is not feasible, we aim to present a short introduction to several effective techniques for modeling creep behavior. Ghoniem et al. [126] are widely recognized for their pioneering contributions to dislocation-based models of creep in engineering materials, particularly those subjected to high temperatures. Their research focuses on the role of dislocations and their dynamics, such as glide and climb, in governing time-dependent deformation. By incorporating microscale mechanisms like dislocation motion and interactions into a macroscale continuum framework, Ghoniem’s models offer a multiscale approach to predicting creep behavior under varying stress and temperature conditions. Their work, applied to high-temperature materials like ferritic–martensitic steels or nickel-based superalloys, enhances the understanding of long-term material performance in extreme environments, such as power plants and aerospace applications.

Building on Ghoniem’s modeling framework, Riedlsperger et al. [127,128] integrated dislocation dynamics with a mean-field approach to describe time-dependent deformation under stress and elevated temperatures. Their models focus on key dislocation mechanisms, such as glide and climb, which dominate creep behavior at high temperatures. The mean-field model simplifies dislocation interactions by averaging collective behavior and tracking the evolution of dislocation density during creep. This approach is grounded in the modified Orowan equation [129,130], where the creep strain rate is expressed as the product of dislocation density, Burgers vector, and dislocation velocity. Central to this framework is the role of dislocation motion in driving creep deformation. Dislocation velocity, influenced by stress and temperature, dictates the material’s response to external loads, while dislocation density evolves through continuous processes of generation, interaction, and annihilation. This multiscale approach links microscale dislocation behavior to macroscale material performance, providing significant insights into the creep behavior of complex materials, including superalloys and steels, in high-temperature environments. Similar methods have already been investigated on IN718. For instance, Wu et al. [131] proposed a model considering GBS-inhibiting particles and nucleation of cavities at GBs, in addition to the dislocation glide and climb processes.

Morch [132] proposed a macroscopic creep modeling framework, presenting a comprehensive approach that integrates various models to capture the complete creep behavior across all three stages of deformation: primary, secondary, and tertiary creep. For the primary stage, Morch employed an isotropic hardening model to describe the initial phase, where the creep strain rate decreases due to strain hardening. During secondary creep, the Norton–Hoff law was utilized to relate the steady-state creep strain rate to the applied stress through a power-law relationship, often incorporating temperature-dependent Arrhenius terms. In the tertiary stage, Morch used the Rabotnov–Kachanov damage model [133], incorporated a damage variable to account for accelerated deformation and eventual failure due to the accumulation of internal damage, such as voids and cracks. This macroscopic approach is more convenient to use at the component scale, and its parameters can be fitted using both experimental tests and virtual tests produced by the aforementioned mean-field method.

## 4. Creep in LPBF IN718

Creep performances of LPBF IN718 are still discussed today due to the absence of consensus regarding optimal microstructures and associated properties. While features like the significance of the Build Direction [8,15,67,91,104,106] and heat treatments [12,15,18,67,91,92] are well-recognized, most characteristics remain poorly understood.

The formation of micro-voids is known to be the basis for the initiation of creep failure, a phenomenon that is achieved due to the coarsening of micro-voids that leads to the formation of cracks. The presence of porosities in AB AM parts remains a major concern. In this respect, HIP applied to AM parts with the aim of ensuring their full densification is a potential solution. Nevertheless, only a limited number of studies have focused on this topic concerning IN718 developed by LPBF. Vilanova et al. [134] studied the effect of HIP on crack healing in IN738LC and demonstrated that this process is effective if a critical internal cracking thickness is not exceeded. However, the microstructure after HIP is significantly changed with massive precipitation of γ′ and a significant increase in hardness. If the authors conclude that such a modification of the microstructure can improve the mechanical properties, none of them had been evaluated, including creep. In their work, Lesyk et al. [135] compared the effects of HIP, shot peening, ultra sonic impact treatment, and combined post-treatments on the microstructure composition and homogeneity, grain size, and both the surface and internal defects of an LPBF IN718, without focusing on mechanical properties. Li et al. [136] studied the effect of thermal post-treatments on the creep behavior (650 °C/700 MPa) of an AM IN718. They considered the subsequent HIP process to achieve full densification within LDED parts. Comparisons with cast and wrought samples then showed an improvement in creep life, but only for the material that underwent homogenization followed by double-aging. By contrast, the material subjected only to HIP after LDED showed poor creep results, below those of cast and wrought specimens. Pignatelli combined HIP with LPBF IN718 to improve creep properties [137]. In his study, optimized temperature and pressure parameters allowed for achieving full densification and grain coarsening. However, his work presented no validation using creep tests.

While AM and especially LPBF of IN718 are largely studied in the literature, creep performances are still understudied. This gap is particularly significant, given that LPBF IN718 is primarily used in high-temperature applications where creep is a critical design consideration. Improving creep resistance requires identifying the microstructural mechanisms at play to predict optimal material features. Wu et al. [17] defined creep resistance through two main characteristics. Firstly, the resistance to creep deformation was identified by the creep rate at steady-state ${\dot{\varepsilon }}_{ss}$. The lower the steady-state creep rate, the higher the hindering of dislocation motion and the higher the resistance to creep deformation. Secondly, the resistance to creep fracture was identified by the time to fracture $t_{f}$. The higher the time to fracture, the lower the cavity nucleation and growth rates and the higher the resistance to creep fracture. To achieve creep resistance, a material must effectively hinder dislocation motion and minimize cavity nucleation and growth rates. Note that several authors reported a clear correlation between the steady-state creep rate and time to fracture. Finally, appropriate heat treatments (HTs) have to be designed to generate a microstructure able to hinder dislocation motion and to prevent cavity nucleation and growth.

### 4.1. Creep Mechanism Identification

**Figure 18:**
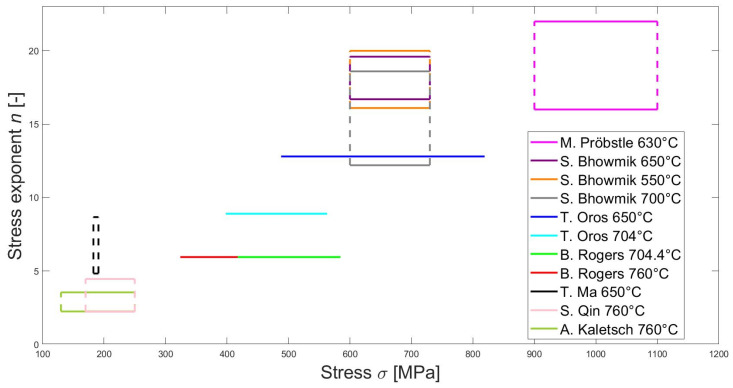
Experimental stress exponent *n* for various ranges of stress.

To begin with, the principal creep deformation mechanism of LPBF IN718 must be identified for various stress–temperature regimes. For this purpose, the stress exponent *n*, as discussed in the previous section, will be considered, acknowledging its potential limitations, as noted by Sandström [124]. A creep exponent around 1 would indicate diffusion creep, while for values ranging from 3 to 8, dislocation creep can be considered as dominant. Note that both mechanisms are expected to always take place with varying importance. Few studies clearly identify the creep mechanism, often due to insufficient experimental data or inconclusive stress exponent values. Indeed, out of 41 papers on the creep behavior of LPBF IN718, only seven explicitly identified a stress exponent. The results are summarized in Table 4 and Figure 18, showing experimental stress exponents for various stress ranges. Note that some authors conducted experiments for different IN718 microstructures, generating different stress exponents for the same stress range. These results are not explicitly differentiated in Figure 18.

A first observation that can be drawn from the literature is that all authors except Oros et al. [102] and Ma et al. [139] explicitly identify the creep deformation mechanism to be based on dislocation. This assumption is always based on the fact that the stress exponent is higher than two, excluding diffusional creep. In addition, no study identifies any transition between a low and a high stress exponent region. Ma et al. [139] did not explicitly identify the creep mechanism, but their range of the stress exponent (4.83–8.67) clearly coincides with dislocation creep. Oros et al. [102] identified the creep mechanism to be in the “Power-Law Breakdown” region, which was defined by Drexler et al. [21] as a region where dislocation creep does not hold due to excessive stress. Instead of climbing over obstacles, dislocations would overcome them by shearing or bowing. This assumption comes from the fact that the stress exponent is larger than 7. However, other authors obtained results in the same range or higher and still considered regular dislocation creep to be the main mechanism. Pröbstle et al. [18] even considered a stress exponent in the range of 16–22 to be in good agreement with dislocation creep in CM materials from the literature. Note that no conclusion can be drawn regarding the impact of temperature on the stress exponent since no systematic study based on a single IN718 microstructure has been performed. It is also interesting to notice that Pröbstle et al. [18] performed compressive tests, allowing for higher stresses and higher stress exponents. Ma et al. [139] performed Small Punch Creep tests, leading to a more intricate identification of the representative stress using a stretch membrane model [141].

A second observation is that the stress exponent seems to increase rather linearly with the applied stress (see Figure 18). This could be explained by the fact that even if no low-stress exponent region has been observed, diffusional creep could play a secondary role in creep deformation. The smaller the applied stress, the higher the relevance of diffusional creep and the lower the stress exponent. Conversely, the higher the applied stress, the smaller the impact of diffusional creep and the higher the stress exponent. Additionally, Wu et al. [17] observed that grain size has a negligible impact on the creep lifetime, potentially indicating that diffusional creep and GBS are not the main creep mechanisms at 650 °C and 650 MPa. McLouth et al. [101] observed planar slip bands at 650 °C and 690 MPa. They interpreted this as an indicator for dislocation creep. Finally, other authors concluded dislocation creep to be the main mechanism for samples manufactured by other AM methods such as Electron Beam Melting (EBM) [142].

As proposed by Rogers [138], a significant difference between CM and AM IN718 resides in the absence, or considerably reduced significance, of diffusional creep and GBS in AM IN718. The causes could be the pinning of GBs by precipitates such as δ or Laves and a lower diffusion in the material, hindering diffusional creep.

### 4.2. Creep Strength

Following Wu et al. [17], creep strength (identified through the minimum strain rate) is, along with creep lifetime, one of the two main creep characteristics. Assuming, from the previous section, that creep deformation is controlled by dislocation motion, one can deduce that the resistance to creep deformation of LPBF IN718 depends on its ability to hinder dislocation motion. As previously mentioned, several strengthening mechanisms exist. Precipitation hardening, solid solution hardening, or strain hardening are the most popular. Additionally, features such as twin boundaries and cellular structures contribute to material strengthening. Using Machine Learning, Sanchez et al. [143] also identified porosity as the most influential parameter affecting the creep rate of LPBF IN718. Finally, at low stress and high temperature where diffusional creep could be considered, an increase in the grain size can provide a strengthening effect, as observed by Chaturvedi et al. [13] on CM IN718. According to the theory of Jones et al. [115] and Ashby et al. [116], this microstructure reduces the density of vacancy sources and sinks (i.e., the grain boundaries) and increases the diffusion distance for atoms (i.e., the distance between grain boundaries).

#### 4.2.1. Precipitation Strengthening

IN718 is known to be a precipitation hardening alloy. Its strength mostly comes from the coherency strains induced by the γ′ and γ″ nanoscale precipitates. The impact of these phases has been largely evidenced in the literature both on AM and CM IN718. Indeed, Chaturvedi et al. [13] performed a systematic study on the impact of coherent γ″ size on the steady-state creep rate of CM IN718 at 600 °C and for stresses ranging from 670 MPa to 815 MPa. They concluded that regardless of the stress, the steady-state creep rate first decreases for increasing particle sizes until reaching a minimum value, after which it starts increasing. The optimal particle size is found to be slightly smaller for a minimum creep rate than for optimal tensile properties at room temperature. Kaletsch et al. [7] also highlighted that excessive coarsening of γ″ phases leads to a decrease in creep strength. In addition to its dependence on precipitate size, the steady-state creep rate depends on the volume fraction of precipitates. This was highlighted by Shi et al. [8], Kaletsch et al. [7], and Wu et al. [17]. The latter observed a decrease in the steady-state creep rate from (1.53 ± 0.27) × 10^−8^ [1/s] to (3.03 ± 0.89) × 10^−9^ [1/s], associated with an increase in the volume fraction of γ″ precipitates from 9.2% to 13%. However, the measured strengthening was higher than predicted by their model, which indicates that it cannot be entirely attributed to precipitation hardening. Consequently, other strengthening mechanisms such as solid-solution hardening, the presence of annealing twins, or hardening by dislocation pile-up at GBs have to be considered. Note that precipitate sizes are mostly defined by the aging process, while the precipitate density depends on the level of Nb homogenization obtained through the solution/homogenization process.

#### 4.2.2. Microstructural Interfaces

According to Section 2, several types of boundaries, such as GBs, subgrain boundaries, and twin boundaries, can be found in LPBF IN718. These microstructural features are usually considered to be beneficial to the creep strength of the alloy. Indeed, they hinder dislocation motion and induce dislocation pile-ups, leading to hardening.

Xu et al. [97] observed that high-angle GBs impede dislocation motion and decrease the creep rate, improving the alloy’s resistance to creep deformation. This observation means that an increased GB density would reduce the steady-state strain rate of the material and improve its creep resistance. This behavior was demonstrated in a systematic way by Chaturvedi et al. [13] for CM IN718. Indeed, they showed that when the material undergoes dislocation creep, decreasing the grain size, i.e., increasing the GB density, reduces the steady-state creep rate. Inversely, they observed that when the material undergoes diffusional creep, increasing the grain size, i.e., decreasing the GB density, reduces the steady-state creep rate, which is coherent with the creep theory in Section 3.1. Note that grain size also impacts creep lifetime due to the high concentration of detrimental phases at GBs, which explains why in practice, even if dislocation creep is the main creep mechanism, most authors consider that large grains are beneficial for creep performances [1,67]. In addition, even if diffusional creep probably plays a small role, large grains can reduce diffusional creep, as observed by Chaturvedi et al. [13].

Based on their model, Wu et al. [17] pointed out that the strengthening effect of γ″ precipitates is not enough to explain the reduced steady-state creep rate that they experimentally observed. They assumed that an additional strengthening effect comes from the hindering of dislocation produced by the highly ordered structure of annealing twins generated during recrystallization. Xu et al. [97] observed similar behavior, concluding that twin boundaries are obstacles to dislocation motion, leading to a decrease in the creep rate.

Finally, substructures are also considered as obstacles to dislocation motion. Indeed, Pröbstle et al. [18] concluded that process-induced subgrains (as opposed to strain-induced subgrains) partially contribute to the superior creep strength of LPBF IN718 compared to CM IN718. Gallmeyer et al. [60] reached the same conclusion. Regarding the material properties at room temperature, Zhao et al. [144] observed that subgrains enhance both the yield strength and ductility of LPBF IN718.

#### 4.2.3. Solid Solution Strengthening

Xu et al. [12] mentioned solid solution hardening as a high-temperature strengthening mechanism for IN718. Note that at room temperature, Zhang et al. [145] demonstrated that this mechanism has a non-negligeable impact on the yield strength of LPBF IN718. Solid solution strengthening of IN718 can then be considered as a hardening mechanism at high and ambient temperatures, but it remains rarely mentioned by authors.

### 4.3. Creep Lifetime

**Figure 19:**
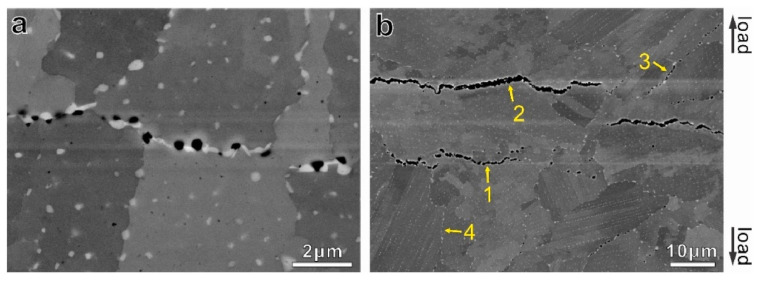
Scanning Electron Microscope–BackScattered Electron (SEM-BSE) images showing (**a**) cavities and (**b**) coalesced cavities forming cracks along GBs nearly perpendicular to the load direction in LPBF IN718 after AMS 5662 heat treatment. Reproduced from [17].

Creep lifetime is primarily governed by void kinetics [17]. Indeed, following the theory developed in Section 3, creep fracture occurs through the growth of cavities into voids, which then coalesce into macroscopic cracks. Cavities form along GBs perpendicular to the applied load due to dislocation accumulation, stress concentration, and atom diffusion. GBs are preferential precipitation areas for detrimental δ and Laves phases, making them weak points and explaining the relevance of grain size and shape to the rupture properties of IN718. In addition, cavity kinetics is also controlled by GBS, which leads to the formation of w-type cracks at the triple points of GBs [12]. Overall, creep fracture in LPBF IN718 is widely recognized as primarily intergranular, as shown in Figure 19. This fracture mechanism is observed by almost every research team [1,12,17,19,103].

#### 4.3.1. GB Particles

Due to their brittleness and incoherent relationship with the matrix, δ and Laves phases, which mostly precipitate at GBs, will be preferred sites for crack initiation, as observed by several authors [1,15,97]. In addition to their effect on crack propagation, those particles retain Nb, which is a necessary element for the formation of strengthening γ″ precipitates. It is still important to note that a few authors consider that δ precipitates might be beneficial for the creep properties of IN718. Indeed, Xu et al. [97] mentioned that a small amount of δ precipitates at the GBs could stabilize the microstructure by preventing grain coarsening, while Sanchez et al. [67] and Xu et al. [12] considered that they can hinder crack propagation along GBs. This point of view remains, however, a minority.

#### 4.3.2. Grain Shape

In addition to the importance of GB density, grain shape is an important characteristic. Indeed, equiaxed grains allow for a reduction in stress concentration through grain rotation [67,97]. Columnar grains inhibit grain rotation and induce high stress concentrations. Since small grains are detrimental to creep lifetime, large equiaxed grains are recommended by Sanchez et al. [19]. In addition, laser overlapping regions lead to small equiaxed grains, displaying high density of detrimental δ and Laves. Consequently, they are prime candidates for crack propagation and should be reduced by an appropriate scanning strategy [67]. Studies also showed that serrated GBs can hinder cavity formation and growth along GBs, largely improving creep lifetime [96,113]. However, inhomogeneous grain growth can lead to regions of high GB density, drastically decreasing the resistance to fracture [96,108].

### 4.4. Optimal Performance–Microstructure–Treatment Relationship

With the key parameters affecting creep strengthening and fracture identified, this section summarizes relevant findings from the literature. As previously discussed, heat treatments will be categorized into Hot Isostatic Pressing (HIP), homogenization and aging (HA), and solution and aging (SA).

For a meaningful comparison, studies involving each heat treatment type under similar test conditions were selected. Tests were performed at 650 °C and between 550 MPa and 750 MPa. Based on the previous subsections, key microstructural features to be taken into account are the size and density of γ″ precipitates, the presence of subgrains and twin boundaries (TBs), the grain size and shape, and the presence of Laves, δ, and carbides at GBs (see Table 5). Time to rupture (TTR) and the steady-state creep rate ${\dot{\varepsilon }}_{ss}$ are the comparison criteria.

Kuo et al. [96] (HTs 7, 11, 17, 18, 20, 26, 29, and 32) performed a large set of heat treatments on LPBF IN718 including HIP, HA, and SA. They observed that the standard AMS 5662 treatment (HT 32) is largely ineffective for LPBF IN718, as it fails to effectively homogenize Nb and promotes the precipitation of detrimental δ and Laves phases at GBs, significantly reducing the rupture strength. They also concluded that increasing the solution temperature to 1045 °C (HT 29), despite improving creep properties, is insufficient to induce recrystallization and dissolution of δ and Laves. An increase in the homogenization temperature to 1065 °C (HT 26) leads to a more than three times larger lifetime since it significantly reduces the concentration of Laves and δ and slightly increases the grain size. Higher homogenization temperatures than 1065 °C (HTs 20, 18, and 17) fully dissolve the detrimental phases but are not able to improve creep performances due to inhomogeneous grain growth. Finally, HIP (HTs 11 and 7) generates even larger grains with serrated GBs and reduces the amount of porosity. When followed by aging (HT 7) to precipitate strengthening phases, this treatment is the most effective post-processing method that has been tested.

Another broad study was performed by Wu et al. [17] (HTs 16, 21, 24, and 33). They observed a slight increase in creep strength and lifetime between AMS 5662 (HT 33) and 1080 °C HA for 1 h (HT 24), likely due to the dissolution of GB δ phases, which extends creep lifetime and probably increases the density of γ″ precipitates. Performances are, however, largely improved after 1080 °C HA for 12 h (HT 21) and 1180 °C HA for 12 h (HT 16), both of which lead to an increase in lifetime by a factor seven and a decrease in creep rate by a factor five compared to AMS 5662. The superior rupture strength can be explained by several factors: grain growth, transformation from columnar to equiaxed grains, and δ dissolution. The reduced creep rate is explained by γ″ growth from 16.6 nm to 17.2 nm, by an increase in γ″ volume fraction of 3.8%, and by the presence of twin boundaries. Note that the higher temperature of HT 16 does not improve the creep performances compared to HT 21, which can be explained by a reduced twin boundary density, mitigating the effect of larger grains.

Similar to the two previous studies, Shi et al. [1] compared a standard 980 °C SA (HT 31) close to AMS 5662 to a 1065 °C HA treatment (HT 15). They observed that the HA sample exhibited a rupture time nearly four times longer than the SA sample. Additionally, its steady-state creep rate is reduced by a factor 6.7. This large improvement in creep properties is associated with the dissolution of GB δ phases, leading to stronger GBs and larger $\gamma^{\prime }/\gamma \prime \prime$ precipitates. The authors also observed grain growth during the creep of the HA sample, which is allowed by the dissolution of δ precipitates that has a pinning effect on GBs. Improved performances are also associated with a lower stress concentration at GBs and a higher dislocation density within grains. As previously mentioned, larger grains are beneficial for creep performance. Note that these tests were performed with the load applied perpendicular to the BD. The same research team later studied the impact of the loading direction. They observed, on the same HA treated sample, that a loading parallel to the BD increases the creep lifetime of the sample by a factor three and significantly reduces the steady-state creep rate [8]. This is because the optimal loading, i.e., parallel to the BD, is the one that reduces the number of GBs perpendicular to the loading.

Finally, Chizari et al. [108] compared the creep properties of LPBF IN718 treated with AMS 5662 (HT 33): 1060 °C HA for 1 h (HT 28) and 1130 °C HA for 1 h (HT 19). Similar to the other studies, they observed that AMS 5662 leads to poorer creep performances than HA. They found that 1060 °C HA improves the time to rupture by a factor 2.6 and decreases the minimum creep rate by a factor 6.55 compared to AMS 5662. This is explained by the dissolution of a large amount of δ accompanied by an increase in both the density and size of γ′/γ″. In addition, carbides remain small, and serrated GBs are observed. However, 1130 °C HA displays worse performances than the previous treatment. This could be explained by carbide coarsening, loss of serrated GBs, and inhomogeneous grain growth, leading to areas of high GB density.

Overall, general trends can be deduced from the experimental campaigns described in Table 5. On the microstructural side, the dissolution of δ phases is critical to liberate Nb for γ″ precipitation and to lose their pinning effect on GBs, allowing grain growth. Similar to Laves phases, δ precipitates are preferential crack initiation sites. In addition, recrystallization and grain growth are beneficial to creep performance and are associated with the formation of twin boundaries, which also improve high-temperature strength. Serrated GBs bring additional crack propagation resistance to the alloy. Minimal density of porosity is also a critical factor. Regarding the heat treatment, an optimal procedure has not been identified, but trends from the literature are clear. To begin with, SA treatments like the classical AMS 5662 standard are largely inefficient for LPBF IN718. Indeed, such low-temperature treatments promote the precipitation of highly detrimental δ phases due to high Nb concentration at grain and subgrain boundaries. Even if they remove beneficial subgrains, homogenization temperatures starting around 1060 °C are required to fully dissolve δ and to promote recrystallization and grain growth. However, excessive treatment time or homogenization temperatures above ≈1180 °C lead to inhomogeneous grain growth and coarsening of detrimental carbides. Finally, HIP treatments facilitate grain growth, porosity reduction, and Laves dissolution. They, however, lead to excessive carbide coarsening and are very expensive in terms of energy and equipment. These microstructural features and associated creep performances are summarized in Table 6.

### 4.5. Comparison Between Optimized LPBF IN718 and Reference Conventionally Manufactured IN718

**Figure 20:**
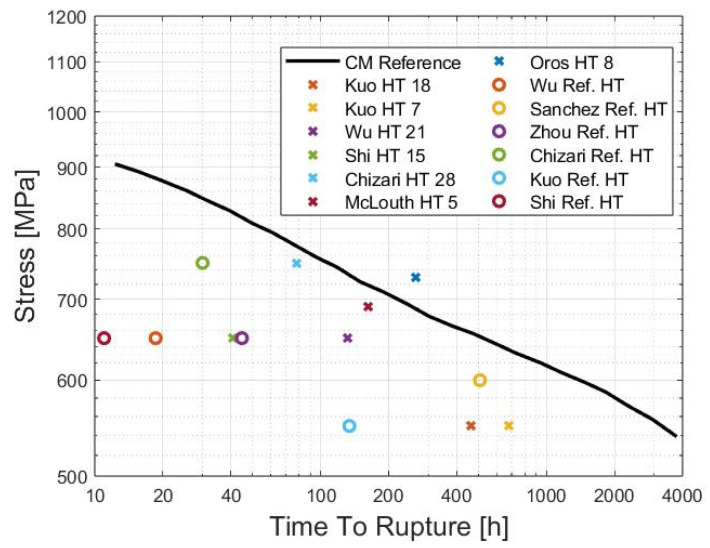
Comparison of TTR for CM IN718 (black line), AMS 5662 HT LPBF IN718 (circled points), and optimized HT LPBF IN718 (crossed points) at ≈650 °C.

Finally, a comparison of the creep performance between the optimized HT LPBF IN718 and the reference CM IN718 is essential for contextualizing the results within the current industry standards. To this end, data sheets [146] gathering the creep properties of hot-rolled IN718 treated following an AMS 5662-type HT were used as a reference. Figure 20 gathers experimental results of creep tests at 650 °C performed on LPBF samples after AMS 5662 HT (circled points) and after optimized HTs (crossed points) as well as a stress–TTR curve obtained from CM samples.

A first observation is that, although the LPBF sample of Oros et al. [102] exhibits a better TTR than the CM samples, all other LPBF samples show worse performance than the CM reference curve. Additionally, except for the sample from Sanchez et al. [67], the AMS 5662 HT LPBF samples are significantly worse than the optimized HT LPBF samples. Finally, while some samples closely follow the CM curve and, in one case, exceed it, their creep performance generally remains below the reference level. Note that among the three best LPBF samples, two underwent HIP treatments (McLouth et al. [101] and Oros et al. [102]) and one went through an HA treatment (Chizari et al. [108]). This behavior is coherent with Section 4.4.

Overall, although significant improvements in the creep properties of LPBF IN718 have been reported in the literature through specifically designed HTs, the standards of CM IN718 are generally not met, indicating the need for further research.

## 5. Conclusions

LPBF IN718 is currently the subject of extensive research, particularly for high-temperature applications in the aerospace, energy, and industrial sectors. However, the creep behavior of LPBF IN718 remains an understudied area, despite its critical importance for the design of high-temperature components. It has yet to meet the standards of CM IN718. Currently, there is no universally accepted HT, and the microstructural features that optimize creep resistance are still debated. Based on the literature, key trends for optimal creep performances include the following:

- High density and optimal size of $\gamma^{\prime }/\gamma \prime \prime$ strengthening precipitates;
- Dissolution of Laves and δ detrimental phases;
- Large equiaxed grains;
- Presence of twin boundaries and subgrains, if possible;
- No excessive carbide coarsening;
- Low porosity.

These key microstructural features can be achieved through homogenization above ≈1060 °C + DA or through HIP, the latter allowing for larger grain growth and porosity decrease. Conventional SA around 980 °C + DA is extremely detrimental to the strength of the alloy due to excessive δ precipitation and inefficient homogenization of segregated alloying elements.

Additionally, the loading direction is a critical factor for creep strength due to the columnar nature of the grains. Consequently, components should be designed such that the loading is applied parallel to the BD. This effect is mitigated by recrystallization when samples are submitted to high-temperature HTs, leading to equiaxed grains.

Certain topics remain open for further investigation. While δ phases are generally considered detrimental, some studies suggest that finely distributed δ phases may stabilize the microstructure. The impact of grain size, although widely discussed, also needs further clarification.

Other factors, such as powder reuse [100] and scan strategy [67], have been investigated but were not covered in this article. Indeed, the manufacturing process itself has a large impact on the AB microstructure and significantly influences the choice of HT for creep optimization. Additionally, the introduction of inoculants such as Yttrium [147,148], or Boron and Phosphorus [149], has shown promise in improving grain structure and mechanical properties.

## Figures and Tables

**Table 1:** Composition range in weight percent for IN718 [3,6,17,18,20,21,22,23].

	Ni	Cr	Fe	Nb	Mo	Ti	Al	C
wt.%	Bal.	18–19.6	17.6–22	4.9–5.4	2.85–3.4	0.9–1.04	0.3–0.72	0.03–0.04

**Table 2:** Comparison of creep lifetime and minimum creep rate between conventionally manufactured samples and Laser Powder Bed Fused As-Built samples for IN718 alloy.

Manufacturing Process	Test Conditions	Creep Lifetime [h]	Minimum Creep Rate [10^−5^ h^−1^]	Reference
AB LPBF + Stress Relief Cast	700 °C/325 MPa	712 2000	/	[91]
AB LPBF Wrought	650 °C/750 MPa	12 143	200 4.06	[12] [93]
AB LPBF Wrought	650 °C/620 MPa	7.03 23	/	[92]
AB LPBF Cast and Wrought	650 °C/550 MPa	270 1200	/	[15]
AB LPBF Wrought	650 °C/500 MPa	107 ± 17 409 ± 8	12.4 ± 1.1 2.47 ± 0.1	[67]

**Table 4:** Experimental studies on the stress exponent used to identify creep mechanisms.

Reference	Temperature [°C]	Stress Range [MPa]	Stress Exponent *n* [-]	Test Type	Identified Creep Mechanism
Pröbstle et al. [18]	630 °C	900–1100	16–22	Compressive	Dislocation creep
Bhowmik et al. [100]	550 °C 650 °C 700 °C	600–730	16.1–20 16.7–19.6 12.2–18.6	Tensile	Dislocation creep
Oros et al. [102]	650 °C 704 °C	488–819 398–563	12.8	Tensile	Other (Power-Law Breakdown)
Rogers et al. [138]	704.4 °C 760 °C	417.5–584.5 324–486	5.95	Tensile	Dislocation creep
Ma et al. [139]	650 °C	182.72–190.94	4.83–8.67	Small Punch Creep	Not explicitly mentioned
Qin et al. [140]	760 °C	170–250	2.23–4.46	Tensile	Dislocation creep
Kaletsch et al. [7]	760 °C	130–250	2.25–3.55	Tensile	Dislocation creep

**Table 5:** Comparison of microstructural features with associated time to rupture (TTR) and the steady-state creep rate ${\dot{\varepsilon }}_{\mathrm{ss}}$. HT refers to the heat treatment numbered in Table 3, and the colors refer to the following heat treatment categories: green for HIP, grey for HA, and yellow for SA. For Laves, δ, and GB carbides, a qualitative symbol describes their relative presence: “-” for small amount, “+/-” for medium amount, “+” for large amount, “++” for very large amount, and “/” for undetected presence. The grain size is described by the same symbols. Subgrains and twin boundaries (TBs) are either present (ticked) or not (unticked). Tests are performed at 650 °C between 550 MPa and 750 MPa.

HT	Ref.	*γ*″ Size [nm]	*γ*″ Volume Fraction [%]	Subgrains	TB	Grain Size	Grain Shape	Laves	*δ*	GB Carbides	TTR [h]	${\dot{\varepsilon }}_{\mathrm{ss}}$ × 10^9^ [1/s]
7	[96]	No data	No data	☐	☒	++	Serrated uneven equiaxed	/	/	++	677	89
11	[96]	No data	No data	☐	☒	++	Serrated uneven equiaxed	/	/	++	493	173
16	[17]	No data	No data	☐	☒	++	Equiaxed	+	/	+	±131.5	≈3.03
17	[96]	No data	No data	☐	☒	++	Uneven equiaxed	/	/	++	151	174
18	[96]	No data	No data	☐	☒	++	Uneven equiaxed	/	/	+	462	137
19	[108]	No data	No data	No data	No data	+	Uneven equiaxed	No data	/	+	≈52	4820
20	[96]	No data	No data	☐	☒	+	Uneven equiaxed	/	/	+	230	159
21	[17]	17.2	13	☐	☒	+	Equiaxed	+	/	+	131.5	3.03
24	[17]	No data	No data	☐	☐	-	Columnar	+	/	+	25.1	11.9
15	[1]	150 by 30	No data	No data	☐	-	Columnar	+	/	No data	≈41	≈75
26	[96]	No data	No data	☐	☐	+/-	Columnar + equiaxed	/	-	+/-	426	190
28	[108]	65.2	No data	No data	No data	-	Columnar + equiaxed	No data	-	+/-	≈78	1060
29	[96]	No data	No data	☒	☐	-	Columnar + equiaxed	+	+	+/-	254	159
31	[1]	100 by 20	No data	☒	☐	-	Columnar	+	+	No data	≈11	≈500
32	[96]	No data	No data	☒	☐	-	Columnar + equiaxed	++	++	/	134	1070
33	[17]	16.6	9.2	☒	☐	-	Columnar	+	+	/	18.6	15.3
33	[108]	33.6	No data	☒	No data	-	Columnar + equiaxed	No data	+	-	≈30	6940

**Table 6:** Summary of the microstructural features and associated impact on creep due to HA (homogenization + aging), SA (solution + aging), and DA (direct aging) heat treatments.

Heat Treatment	Microstructural Effect	Impact on Creep Behavior
**HA** Homogenization (1060–1180 °C) + Aging (620–760 °C)	Recrystallization, grain growth, and presence of twin boundaries. Loss of substructure. Nb and Ti homogenization. Increased amount of *γ*′/*γ*″ precipitates. Absence of *δ* phase. Decreased amount of Laves phase.	Large grains enhance the resistance to void nucleation and crack propagation. The large density of *γ*′/*γ*″ precipitates improves the resistance to dislocation motion. The absence of detrimental phases increases the resistance to fracture.
**SA** Solution (850–1060 °C) + Aging (620–760 °C)	Neither recrystallization nor grain growth. Insufficient homogenization of Nb and Ti. Insufficient precipitation of *γ*′/*γ*″. Extensive precipitation of *δ* phase.	Small grains promote the nucleation of voids. The excessive precipitation of *δ* and Laves phases is detrimental to the crack resistance. The low density of *γ*′/*γ*″ precipitates reduces the dislocation motion resistance.
**DA** Direct Aging (620–760 °C)	Neither recrystallization nor grain growth. No Nb and Ti homogenization. Substructure retained. Largely insufficient precipitation of *γ*′/*γ*″. Precipitation of *δ* phase depends on the aging temperature and time.	Small grains promote the nucleation of voids. Laves phases are detrimental to the crack resistance. The low density of *γ*′/*γ*″ precipitates reduces the dislocation motion resistance. Substructures increase the dislocation motion resistance.
